# Ferroptosis in health and disease

**DOI:** 10.1186/s43556-026-00637-8

**Published:** 2026-10-24

**Authors:** Precious Adeoye Oyedokun, Victory Jesutoyosi Ashonibare, Tunmise Maryanne Akhigbe, Precious Jesutofunmi Ashonibare, Adetomiwa Ezekiel Adeogun, Cecilia Adedeji Adegbola, Ayomide Isaac Omole, Roland Eghoghosoa Akhigbe

**Affiliations:** 1 Department of Physiology, Ladoke Akintola University of Technology, Ogbomoso, Oyo State, Nigeria; 2 Reproductive Biology and Toxicology Research Laboratory (ReBTReL), Oasis of Grace Hospital, Osogbo, Nigeria; 3 Cardiovascular Regenerative Medicine & Tissue Engineering 3D Lab, Department of Cardiovascular Surgery and Research Group for Experimental Surgery, Heinrich Heine University, Medical Faculty, Düsseldorf, Germany; 4 Breeding and Genetic Unit, Department of Agronomy, Osun State University, Osun State, Nigeria; 5 Department of Physiology, Babcock University, Ilishan Remo, Ogun State Nigeria

**Keywords:** Ferroptosis, Glutathione, Lipid peroxidation, Iron, Oxidative stress

## Abstract

Ferroptosis is an iron-dependent, non-apoptotic form of regulated cell death that is driven by lipid peroxidation. In the past decade, it has emerged as a core pathogenic mechanism in a wide range of human diseases, such as neurodegenerative disorders, cardiovascular disease, cancer, and notably, reproductive dysfunction. This review provides a comprehensive overview of the molecular mechanisms underlying ferroptosis with a specific focus on the System Xc⁻–GSH–GPX4 axis, iron metabolism, lipid peroxidation, and the regulatory roles of key modulators such as Nrf2, ACSL4, and CISD2. This review also explores the physiological roles of ferroptosis in development, tissue homeostasis, and immune regulation, as well as its pathological contributions to obesity, diabetes mellitus, atherosclerosis, neurodegeneration, stroke, cancer, infectious diseases, and reproductive disorders. A substantial part of this review discusses the role of ferroptosis in the pathogenesis of reproductive disorders, such as male reproductive pathologies, including varicocele, testicular ischemia–reperfusion, cryptorchidism, oligospermia, erectile dysfunction, and testicular cancer, and female pathologies like primary ovarian insufficiency, polycystic ovary syndrome, endometriosis, ovarian cancer, and pre-eclampsia. Moreover, the interplay between ferroptosis and immune dysfunction in reproductive tract infections was highlighted. Furthermore, we evaluated current biomarkers, emerging therapeutic strategies, and key translational challenges, highlighting the promise of ferroptosis-modulating agents for novel diagnostic and therapeutic interventions in reproductive medicine and beyond.

## Introduction

Ferroptosis is an intriguing and relatively new discovery in cell death mechanisms. It was identified in 2003 as a different form of traditional cell death, such as necrosis and apoptosis, with unique characteristics and dependencies. The term "ferroptosis" was introduced by Dr. Brent R. Stockwell in 2012. Ferroptosis is a novel iron-dependent form of cell death distinct from caspase activation or cytochrome c release but characterized by lipid peroxidation [1]. This iron-centric process shows an important interaction between cellular iron metabolism and reactive oxygen species (ROS), fundamentally changing our knowledge of cell death pathways. Although oxidative stress and inflammation have been associated with the pathogenesis of human diseases for years [2–4], ferroptosis has emerged as a central mediator in the pathogenesis of reproductive diseases [5–8] and several other human diseases like cancer [9], neurodegeneration [10], and cardiovascular disease [11].

The review explores the mechanistic pathways underlying ferroptosis, focusing on the intricacies of iron homeostasis and the System Xc-GSH-GPX4 pathway. It highlights the role of iron dysmetabolism in cellular alterations, particularly lipid peroxidation, which serves as a hallmark of ferroptotic cell death. The review also highlights different regulatory mechanisms that control intracellular iron levels, such as the heat shock protein 1 (HSPB1) and iron response element binding protein 2 (IREB2). Furthermore, the review highlights the role of ferroptosis in physiological and pathological conditions. By integrating current scientific understanding with developing therapeutic techniques targeting ferroptosis, this review provides a broad picture of a pathway that holds promise for creative therapies in many disorders. The emphasis on biomarkers for the detection and modulation of ferroptosis further demonstrates its potential for therapeutic applications in medicine, underscoring the necessity for ongoing research in this promising area.

## Mechanisms of ferroptosis

**Fig. 1:**
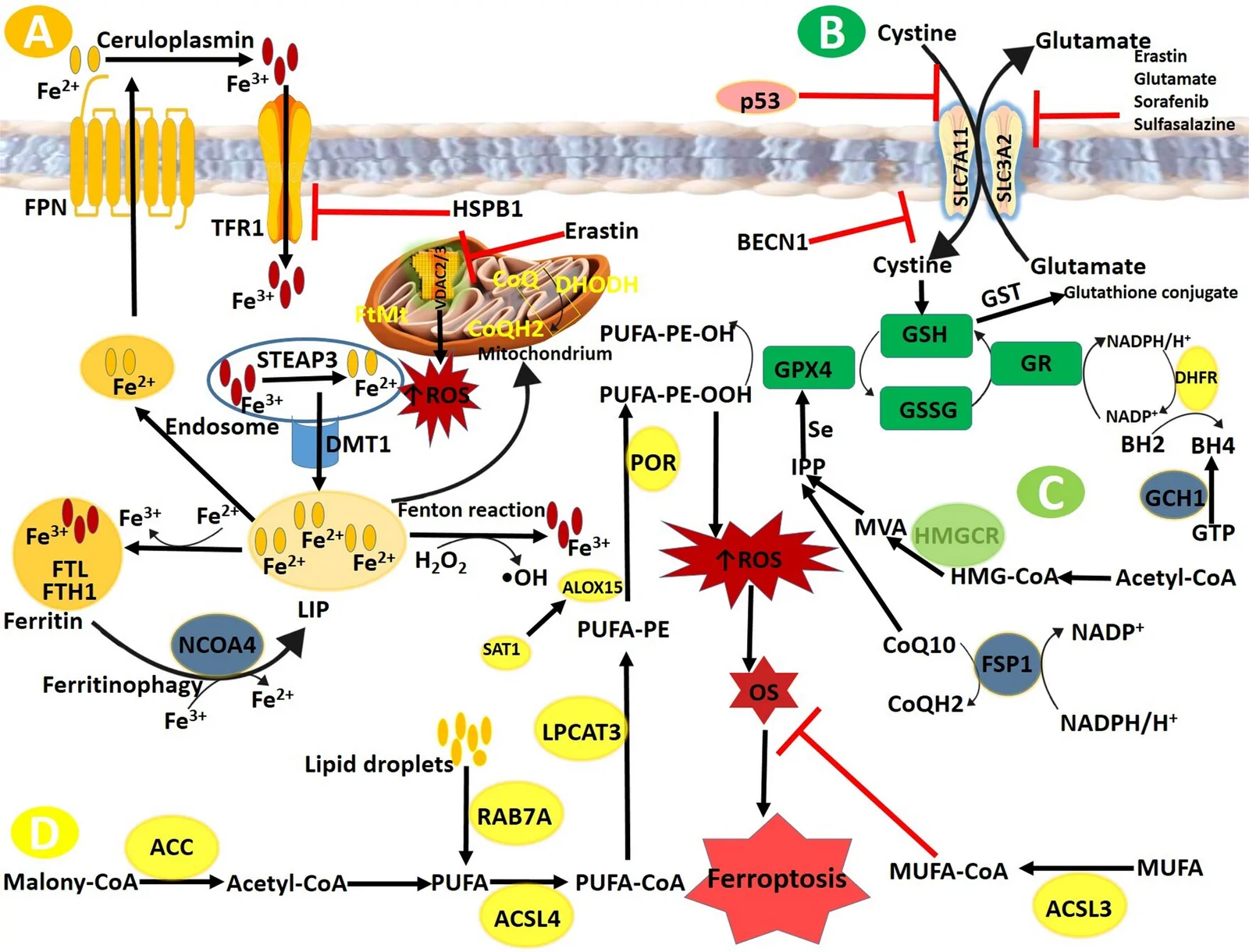
Mechanisms associated with the initiation of ferroptosis. **A** Ferroptosis results from disrupted iron metabolism. Normally, transferrin-bound Fe3⁺ enters cells via TFR1, and it is reduced to Fe2⁺ by STEAP3, and joins the labile iron pool for storage, export, or mitochondrial use. NCOA4-mediated ferritin degradation releases excess Fe2⁺, driving ROS accumulation, lipid peroxidation, and ferroptosis; HSPB1 counteracts this by suppressing TFR1. **B** The System Xc–GSH–GPX4 pathway controls ferroptosis by regulating cystine uptake and glutathione metabolism. SLC7A11/SLC3A2 import cystine for GSH synthesis, while GPX4 uses GSH to detoxify lipid hydroperoxides, preventing lipid peroxidation. Inhibition of this system disrupts redox balance, increases ROS, and triggers ferroptosis; inhibitors include BECN1, p53, and erastin. **C** HMG-CoA reductase (HMGCR) converts HMG-CoA to mevalonate, producing IPP essential for GPX4 and CoQ10 synthesis. FSP1 prevents ferroptosis by converting CoQ10 to its antioxidant form CoQ10H₂ via NADPH. Additionally, BH₄, generated by GCH1 or DHFR, scavenges free radicals and enhances glutathione antioxidant activity, preventing lipid peroxidation and ferroptosis. **D** Polyunsaturated fatty acids (PUFAs) promote lipid peroxidation and ferroptosis. ACC and lipophagy generate PUFAs, which are converted by ACSL4 and LPCAT3 to PUFA-PE. ALOX15, POR, and hydroxyl radicals oxidize PUFA-PE to toxic PUFA-PE-OOH, detoxified by GPX4. GPX4 depletion increases ROS and ferroptosis, while MUFA-CoA (via ACSL3) prevents it

Ferroptosis is a type of regulated cell death that differs from other cell death types. It is characterized by iron-dependent lipid peroxidation and oxidative stress. Unlike apoptosis or necrosis, ferroptosis involves distinct molecular pathways (Fig. 1) and biomarkers, making it a noteworthy focus of current biomedical research. An in-depth knowledge of the interconnected complex mechanisms, detection methods, and potential therapeutic interventions related to ferroptosis is essential, as this process plays a key role in several diseases, such as neurodegeneration, cancer, and reproductive disorders.

### Iron metabolism and System Xc-GSH-GPX4 pathway

The various forms of death that were identified during the previous decades include apoptosis, autophagy, and pyroptosis. In 2003, it was noted that the inhibition of NRAS-mutant HT-1080 cells by Erastin and RAS-selective lethal 3 (RSL3) occurred without the release of cytochrome c, caspase activation, and fragmentation of chromatin in human foreskin fibroblasts. That mechanism had remained unexplained until 2012, when Dr.Brent R.Stockwell showed that the inhibition of NRAS-mutant HT-1080 by erastin was mitigated by iron chelation and named the “iron-dependent form of cell death” called "ferroptosis" [12, 13].

Ferroptosis is initiated by iron metabolism deregulation, leading to iron buildup and inactivation of the System Xc^−^-GSH-GPX4 pathway, leading to reduced cellular cysteine. Under physiological conditions, a balance exists between the uptake of iron and its distribution, storage, utilization, and elimination. Transferrin (TF) is essential in the transport of iron. Ferric (Fe^3+^)-carrying TF binds to transferrin receptor 1 (TFR1) to form a complex that is transported through endocytosis into the cell endosome, where it is reduced to ferrous (Fe^2+^) by six-transmembrane epithelial antigen of the prostate 3 (STEAP3). Fe^2+^ is then transported by divalent metal transporter 1 (DMT1) or zinc transporter 8/14 (ZIP8/14) to form a labile iron pool (LIP) [14]. The iron in this pool may be transported out of the cell through ferroportin (FPN), utilized in the mitochondria (where it is used for the biosynthesis of cofactors necessary for the function of enzymes involved in oxidation–reduction reactions, DNA synthesis, and repair, and a range of other cellular processes), or stored in ferritin, which is made up of Fe^3+^, ferritin light chain (FTL), and ferritin heavy chain 1 (FTH1). However, nuclear receptor co-activator 4 (NCOA4) may induce ferritinophagy (degradation of ferritin), leading to the release of a huge amount of Fe^2+^ and iron overload, thus promoting the Fenton reaction, ROS accumulation, lipid peroxidation, and ferroptosis [15]. Several factors also play vital roles in regulating intracellular iron levels, thus modulating the pathways that lead to ferroptosis. Heat shock protein family B member 1 (HSPB1) inhibits TFR1 expression, leading to reduced uptake of cellular iron and inhibition of ferroptosis [16]. In addition, iron response element binding protein 2 (IREB2) may upregulate FTL and FTH1 and block erastin-induced ferroptosis [17]. Moreover, the Xc-GSH-GPX4 pathway regulates ferroptosis. The Xc-GSH-GPX4 system consists of an amino acid transporter, ubiquitously present in phospholipid bilayer membranes, of the solute carrier family 7, member 11, and the solute family 3, member 2, which drives the cysteine-glutamate exchange; hence, it triggers an enhanced efflux of glutamate along with the excretion of cysteine, the molecule that promotes reduced glutathione (GSH) synthesis, a structural constituent of the glutathione buffer system.

Glutathione peroxidase 4 (GPX4) oxidizes glutathione (GSH) to yield oxidized glutathione (GSSG), which in turn is reduced back to GSH by glutathione reductase (GR) or conjugated with glutathione-S-transferase (GST) [18]. On the other hand, GPX4 usually oxidizes GSH while concurrently reducing cytotoxic lipid hydroperoxides (L-OOH) into lipid alcohols (L-OH) to maintain appropriate redox homeostasis and prevent lipid peroxidation and ferroptosis [19]. Suppression of System Xc-GSH-GPX4 suppresses both cystine influx and GPX4 synthesis, leading to the intracellular accumulation of L-OOH, a cytotoxic molecule that reacts with Fe2 + via a Fenton reaction. The interaction between these creates excessive ROS and hydroxyl radicals•OH, which promote lipid peroxidation and ferroptosis [20]. Common inhibitors of the system Xc-GSH-GPX4 are beclin 1 (BECN1), erastin, glutamate, p53, sorafenib, and sulfasalazine.

Specifically, solute carrier family 40 member 1 (SLC40A1) amplifies FPN activity, leading to greater cellular iron efflux and ferroptosis. The transcription factor nuclear factor erythroid-related factor 2 (Nrf2), which controls antioxidant signaling and action [21], influences ferroptosis through transcription of heme-oxygenase 1 (HO-1) [22], FTH1 [23], and cytoprotective glutathione metabolism genes [24, 25].

### Lipid metabolism and oxidative stress

The transsulfuration, mevalonate, FSP1-CoQ10-NADPH, and GCH1-BH4-DHFR signaling pathways regulate the glutathione system. Under oxidative stress, methionine is converted by transsulfuration to cysteine, which in turn promotes GSH synthesis and dampens oxidative stress-mediated ferroptosis [26]. Also, 3-hydroxy-3-methylglutaryl CoA reductase (HMGCR) converts 3-hydroxy-3-methylglutaryl CoA (HMG-CoA), which is obtained from acetyl-CoA, to mevalonic acid (MVA) that is in turn transformed into isopentenyl pyrophosphate (IPP) that is essential for GPX4 and coenzyme Q10 (CoQ10) synthesis [27]. Additionally, ferroptosis suppressor protein 1 (FSP1), also referred to as apoptosis-inducing factor mitochondria-associated 2 (AIFM2), positively controls mitochondrial apoptosis and inhibits ferroptosis via an apoptosis-independent mechanism. FSP1 may be translocated to the cell membrane, where it undergoes myristoylation and promotes CoQ10 recruitment, and is converted via reduction under the action of NADPH to CoQ10H2, which directly inhibits lipid peroxidation and ferroptosis. Besides, tetrahydrobiopterin (BH4) scavenges free radicals and enhances the antioxidant activities of the glutathione system, thus preventing lipid peroxidation and ferroptosis [28]. BH4 may be produced by the hydroxylation of guanosine triphosphate (GTP) by GTP cyclohydrolase 1 (GCH1) or the reduction of dihydrobiopterin (BH2) (DHFR) by dihydrofolate reductase under the influence of NADPH [27, 29].

Mitochondrial function also plays a critical role in ferroptosis. The mitochondria are enveloped by two membranes. The outer mitochondrial membrane comprises voltage-dependent anion channels (VDACs) that exist in three isoforms: VDAC1, VDAC2, and VDAC3. VDACs play an essential role inthetransport ofions and metabolites.

Erastin may alter VDAC permeability by opening the channels, particularly VDAC2 and VDAC3, leading to the breakdown of the mitochondrial membrane, mitochondrial failure, ROS accumulation, and ultimately ferroptosis.

Besides the above channels, the inner mitochondrial membrane also contains dihydroorotate dehydrogenase, DHODH, involved in the co-extraction of CoQ10 into CoQ10H2, enabling normal redox control and ferroptosis inhibition [30]. Also, mitochondria modulate ferroptosis through the tricarboxylic acid cycle regulation [31]. Cells are prone to lipid peroxidation due to the presence of polyunsaturated fatty acids (PUFAs), which serve as the primary substrate for lipid peroxidation. PUFAs are obtained from dietary sources, acetyl-CoA carboxylase, or lipophagy. ACC catalyzes the conversion of malonyl-CoA to acetyl-CoA and then into PUFAs. Lipophagy of the lipid droplet may be mediated by RAB7A, thus promoting the synthesis of PUFAs. Then, PUFA is converted by ACSL4 to PUFA-CoA and then to PUFA-PE by Lysophosphatidylcholine acyltransferase 3 (LPCAT3). SAT1-driven lipoxygenase (ALOX15), in the presence of cytochrome P450 oxidoreductase (POR) and hydroxyl radicals from the Fenton reaction, then promotes the peroxidation of PUFA-PE to PUFA-PE-OOH, a highly cytotoxic molecule that is detoxified by GPX4 to PUFA-PE-OH [32]. However, when GPX4 is depleted, PUFA-PE-OOH enhances ROS generation and results in lipid peroxidation, which induces ferroptosis [33]. On the other hand, monounsaturated fatty acid (MUFA) may be converted by ACSL3 to MUFA-CoA, preventing ferroptosis via suppression of lipid peroxidation.

### CDGSH Iron Sulfur Domain 2 (CISD2)

In the pathophysiology of reproductive diseases, CISD2 (CDGSH Iron Sulfur Domain 2) contributes to ferroptosis via several pathways besides oxidative stress and lipid peroxidation. CISD2 is necessary for mitochondrial function, iron homeostasis, and cellular defenses. Dysfunction in CISD2 leads to mitochondrial iron overload that blocks energy synthesis and triggers ferroptosis [13, 34]. This mitochondrial breakdown is associated with metabolic dysfunctions in disorders such as endometriosis and PCOS. Moreover, CISD2 regulates iron levels in cells, and its disruption increases iron levels, making cells vulnerable to ferroptosis [35, 36]. Iron overload and inflammation have also been linked to reproductive disorders [37, 38]. CISD2 also modulates autophagy, and impaired autophagy drives ferroptosis with accumulation of damaged organelles and iron overload [39]. CISD2 maintains ER integrity as well, and ER stress triggers ferroptotic pathways that are implicated in reproductive disorders like preeclampsia [40]. CISD2-regulated inflammation also promotes ferroptosis; chronic inflammation (a common complication of most reproductive disorders) can lead to ferroptotic cell death [41]. All these processes demonstrate the complexity of CISD2 in ferroptosis regulation and present promising therapeutic targets to prevent these diseases.

## Ferroptosis in physiology: essential roles in health

Ferroptosis plays a central role in the pathogenesis of diseases and in several physiological processes. It is essential for embryonic development and cell differentiation [42, 43], such as red blood cell maturation through the elimination of immature cells. It also maintains tissue homeostasis by removing damaged cells with high iron and lipid peroxides, thus preventing inflammation and promoting repair [44, 45]. Furthermore, ferroptosis regulates the immune system by controlling immune cell survival and function [46–48]. It acts as a metabolic sensor, linking iron and lipid metabolism to oxidative stress, which has implications for diseases like diabetes and obesity [49].

### Development and differentiation

Ferroptosis plays a key role in the embryonic development and differentiation of various cell types. This is mainly attributed to its induction of selective elimination of either redundant cells, which have no function within the developing organism, or cells capable of interfering with or inhibiting the normal tissue development [42]. In embryonal erythropoiesis, ferroptosis positively regulates the maturation of erythrocytes, or red blood cells, by facilitating the elimination of nucleated immature red blood cells. The elimination occurs through a highly controlled iron-dependent process and involves lipid peroxidation [43]. This regulated cellular apoptosis, to a large extent, affects hematopoietic system formation, thus establishing a physiological balance of the cell populations during organogenesis [43]. The involvement of the iron-carrying protein ferritin and enzyme GPX4 indicates the paramount importance of the maintenance of iron homeostasis and oxidative stress regulation in cell differentiation.

In various developmental processes, ferroptosis is primarily triggered by iron-dependent peroxidation of lipids. In this biological process, the intracellular ferrous iron (Fe2⁺) plays an essential role, as it catalyzes the formation of ROS through a chemical reaction known as the Fenton reaction. The catalytic action induces the peroxidation of PUFAs, which are key components of membrane phospholipids [50]. The accumulation of lipid hydroperoxides compromises cellular membrane, ultimately leading to cell death. GPX4 detoxifies these lipid peroxides through GSH; hence, loss of GPX4 activity leads to ferroptosis [50]. Ferritinophagy, mediated by NCOA4, also releases iron from ferritin to expand the labile iron pool and sensitize cells to ferroptosis. This highly regulated process facilitates effective cell turnover during differentiation [51].

### Maintenance of tissue homeostasis

Ferroptosis plays an essential role in maintaining tissue homeostasis through the effective removal of cells that have accumulated high levels of iron and lipid peroxides, which could otherwise lead to chronic inflammation or tissue damage [44]. This process is triggered by peroxidation of PUFAs in phospholipids of the cell membranes, which is catalyzed by the presence of iron [44]. This peroxidation leads to the formation of lipid hydroperoxides, which subsequently disrupts the integrity of the cell membranes and ultimately leads to cell death. This complex process is tightly regulated by a series of antioxidant mechanisms, especially GPX4, which is important for the reduction of lipid hydroperoxides [50]. When cellular antioxidants are depleted, ferroptosis ensures the removal of aberrant or dysfunctional cells, thus playing a role in the maintenance of overall tissue function [45].

Intracellular labile iron in the ferrous state catalyzes Fenton reaction to generate highly reactive hydroxyl radicals, which are significant in initiating lipid peroxidation [52]. Furthermore, lipoxygenases facilitate and increase the formation of lipid peroxides, particularly on PUFAs. GPX4 reduces lipid hydroperoxides to innocuous substances through the action of GSH, thereby effectively preventing the occurrence of ferroptosis [52]. The breakdown of iron storage systems through ferritinophagy leads to elevated cellular levels of free iron, which further enhances lipid peroxidation and ultimately causes ferroptosis [53]. This biological process can also be observed in bone cells, where ferroptosis plays a role in the control of the viability of both osteoblasts, the bone-building cells, and osteoclasts, the bone-degrading cells. Additionally, in the liver, the process plays a role in hepatocyte turnover and in the formation of fibrosis. Lastly, in the kidney, ferroptosis plays a role in the elimination of damaged tubular cells, ensuring cell repair and regeneration [54–56].

### Immune system function and regulation

Ferroptosis is actively involved in immune regulation by controlling both the survival and the function of various immune cells via inflammation [46]. More precisely, in specialized immune cells such as macrophages and dendritic cells, ferroptosis plays a role in both their polarization and the secretion of cytokines, which is required for the effective clearance of pathogens and tissue repair [47]. Through the removal of excess or dysfunctional immune cells from the body, ferroptosis prevents inflammation and autoimmunity; thus, maintaining immune homeostasis [48].

Immune cell ferroptosis is iron-dependent and mediated by lipid peroxidation. Elevated intracellular iron enhances ROS production, leading to the peroxidation of membrane PUFAs [50]. GPX4, along with glutathione-dependent mechanisms, plays a role in the reversal of lipid peroxidation effects, which is accountable for the regulation of immune cell death. Furthermore, it has been demonstrated that ferroptotic immune cells may release damage-associated molecular patterns (DAMPs) along with oxidized lipids. These molecules play a critical role in immune signaling pathways, thus regulating the secretion of cytokines and the recruitment of immune cells to areas of need [57]. This process is complex and takes place under a fine-tuned equilibrium that exists between immune system stimulation and the induction of tolerance within the body [58, 59].

### Systemic metabolic integration

Ferroptosis establishes a connection between the metabolism of various organs by effectively coordinating the mechanisms of iron metabolism, lipid peroxidation, and oxidative stress protection mechanisms in these organs. The catalytic iron, in turn, catalyzes ROS generation and promotes the occurrence of lipid peroxidation [59]. Lipid peroxidation occurs with PUFAs selectivity in membranes, but GPX4 is utilized to reduce the likes of peroxides through GSH, whose biosynthesis depends on amino acid metabolism [49]. The disruption of these processes increases the vulnerability of cells to ferroptosis, impacting metabolic tissues.

In pancreatic beta cells, ferroptosis has a detrimental impact on insulin secretion due to the disruption in the function of the cell membrane and mitochondria, ultimately leading contributing to incident diabetes [49]. In adipose tissues as well, ferroptosis negatively affects lipid storage capacity and adipocyte survival, demonstrating an association between ferroptosis, obesity and metabolic syndrome [60–62]. Therefore, it is evident that ferroptosis is both a metabolic effector and sensor that efficiently coordinates and responds to cell stress induced by oxidative stress and nutrient status.

## Ferroptosis in various diseases

Ferroptosis is a key factor in various pathologies. In metabolic disorders, iron overload and subsequent ferroptosis in various organs contribute to insulin resistance. In cardiovascular and neurodegenerative diseases, ferroptosis induces cell death in plaques and neurons, respectively. Its role in infectious diseases and cancer is "double-edged," sometimes promoting pathogen clearance, while it may also promote tissue damage that worsens the infection. For cancer biology, ferroptosis seems to be a promising anti-tumor strategy, although cancer cells may evade this pathway. This section provides the in-depth mechanistic pathways linking ferroptosis and the pathogenesis of diseases.

### Metabolic disorders

#### Obesity

Obesity is the accumulation of excess body fat with a harmful impact on health [63, 64]. A body mass index (BMI) over 25 kg/m^2^ is considered overweight, and over 30 kg/m^2^ is obese [63, 64]. In 2015, according to the Encyclopedia of Mental Health, the prevalence of obesity has continued to increase and covers about 100 million children to 600 million adults worldwide, representing approximately 39% of the world's population [65, 66]. Obesity can be a part of a trend because obesity disrupts reproductive function through multiple mechanisms, including a negative impact on spermatogenesis via many different pathways, such as hormonal imbalances, elevated levels of inflammatory mediators, ROS, and testicular heat. [67].

Increased iron level, which is associated with iron metabolism disorders, is directly associated with obesity [68]. Ferroptosis following iron overload is accompanied by increased ROS, inflammatory response, and insulin resistance, causing metabolic disturbances and the incident obesity [69]. Obesity is associated with fat accumulation that decreases glucose tolerance and increases insulin secretion [70]. Also, long-term high-fat diet could lead to iron accumulation and promote the onset of obesity [71]. Available data sufficiently show that in obese individuals, iron accumulation is elevated in the liver, pancreas, and heart, which are typically iron-depleted tissues. The strong correlation between macrophages and iron excess in adipose tissues is largely due to the role of macrophages in regulating iron metabolism [72]. High-fat diet (HFD) promotes fat accumulation, increases iron penetration into adipocytes, decreases the iron-processing ability, and activates M1 macrophage (M1) polarization that in turn accelerates iron accumulation [72–74], demonstrating adipocyte-iron connection. The role of ferroptosis in obesity-induced male infertility suggests that oxidative stress, lipid peroxidation, and iron overload-mediated ferroptosis are all highly correlated in most male reproductive pathologies; reduced GPX4 activity causes lipid peroxidation that leads to ferroptosis [74]. Ferroptosis has also been implicated in placenta disorders, contributing to the etiopathophysiology of pre-eclampsia [75].

#### Diabetes mellitus

Diabetes (DM) is an endocrine disorder resulting in excessively high blood glucose caused by absolute or relative lack in insulin production [63, 76]. The overall prevalence is roughly 1.5 million deaths per year due to undiagnosed or underdiagnosed diabetes [63]. There are now approximately 8.8% of adults (463 million) who are estimated to be suffering from diabetes [77], with male and female infertility as associated complications [78, 79].

Ferroptosis has been shown to play a role in the development, progression, and management of type 2 diabetes mellitus. [80, 81], mainly by promoting islet cell dysfunction and peripheral insulin resistance [80]. Some studies have attributed this to the lack of cellular antioxidant enzymes like superoxide dismutase (SOD), glutathione peroxidase (GPX), and catalase in the pancreas [82, 83]; thus, the susceptibility to incident T2DM increases with a rise in iron store [84, 85]. This is associated with reduced glucose oxidation in the muscle that leads to insulin resistance [86, 87]. Iron deficiency and its excess may influence glucose regulation, and hyperglycemia induces iron overload [88], causing ROS accumulation and ferroptosis. The low-grade inflammatiory state associated with hyperglycemia is also associated with reduced concentrations of H2O2-detoxifying enzymes such as catalase, glutathione peroxidase [89], GPX7, and GPX8 [90], as well as increased ROS production and iron accumulation [91, 92].

In diabetic male, the sperm plasma membrane and acrosome reaction are impaired, contributing to diabetes-induced male infertility [93]. DM patients become hypogonadal at the central (hyperleptinemia or alteration in hypothalamic GnRH pulsatile secretion in the overweight or obese patient) and peripheral (derangement in Leydig cell function) levels of blood gonadotropin and testosterone [94]. Moreover, in diabetic female, the hypothalamic-pituitary-ovarian (HPO) axis may be dysfunctional. Menstrual abnormalities are more common in diabetic women with low BMI [95]. There is increased catabolism in dietary restriction, causes intracellular starvation, particularly before diagnosis and insulin therapy [96] and reduced gonadotrophin-releasing hormone (GnRH) and gonadotropin secretions [96].

### Cardiovascular disease and atherosclerosis

**Fig. 2:**
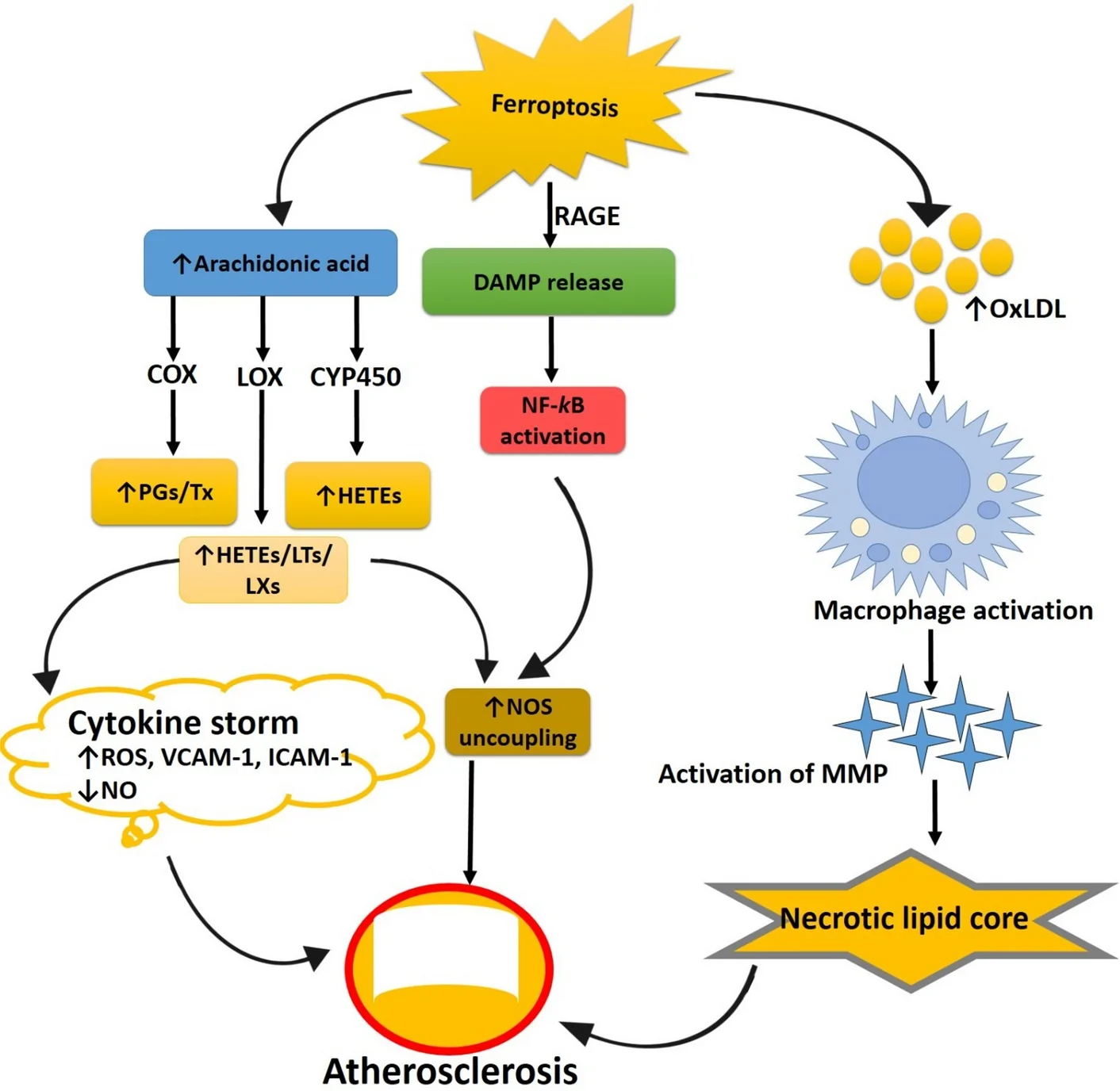
The role of Ferroptosis in atherosclerosis formation. Ferroptosis promotes a rise in the accumulation of arachidonic acid, an unsaturated fatty acid that is released from the cell membrane in response to lipid peroxidation. These fatty acids are metabolized by cyclooxygenases (COX), lipoxygenase (LOX), and cytochrome P450 (CYP450) to generate prostaglandins (PGs), hydro xyeicosatetraenoic acids (HETE) and hydroperoxyeicosatetraenoic acids (HPETE), leukotrienes (LTs), and lipoxins (LXs), which upregulate pro-inflammatory cytokines and promote nitric oxide synthase (NOS) uncoupling. Ferroptosis also promotes the release of damage-associated molecular patterns (DAMPs) from dying cells which activates nuclear factor-kappa B (NF-kB) via the receptor for advanced glycation end products (RAGE). Activated NF-kB promotes the transcription of pro-inflammatory cytokines, thus contributing to cytokine storm and NOS uncoupling. Ferroptosis also enhances the oxidation of low-density lipoprotein (LDL), resulting in increased oxidized LDL (OxLDL) formation, and activation of macrophage matrix metalloproteinase (MMP) with resultant necrotic lipid core formation. These events culminate in atherosclerosis

The pathogenesis of atherosclerosis, a chronic inflammatory disorder characterized by the accumulation of atheromatous plaques in the walls of the arteries, and eventually all forms of acute and chronic clinical CVDs by luminal narrowing and/or thrombosis [97–99], involves iron overload, reduced GPX4, and increased ROS [99, 100]. It was reported that upregulation of GPX4 inhibits the development of atherosclerosis by attenuating lipid peroxidation and the sensitivity of vascular cells to oxidized lipids [101], which otherwiae could cause endothelial impairment (Fig. 2).

Interestingly, the implications of ferroptosis in atherosclerosis extend to biomedical and clinical research with therapeutic considerations [102]. Emerging evidence from recent studies suggests that targeting pathways involved in ferroptosis may be beneficial in the management of atherosclerosis [103–107]. In patients with hemochromatosis, iron restriction therapy lowers atherogenicity [98]. Deferasirox, an iron chelator, attenuated vascular iron deposit, inflammation, and aortic plague deposits in apoE-/- mice [100]. On the other hand, Marques et al. [108] injected iron-dextran into atherosclerotic mice and found that iron overload exacerbated aortic iron deposits, causing endothelial dysfunction via the induction of oxidative stress and an imbalance between the relaxing and contractile factors released by the damaged endothelium.

Moreso, Tongxinluo (TXL), a Chinese medicine that is multifunctional delays the progression of atherosclerosis and reduces cardiovascular events by protecting microvascular barrier and attenuating ferroptosis [102], while Icariin, a principal active component of traditional Chinese medicine *Epimedium grandiflorum Maxim* increased vascular viability and mitochondrial membrane potential while reducing endothelial atherosclerotic plaque and collagen fibre deposits by attenuating oxidative stress and ferrptosis of the vascular endothelial cells [109]. Hydroxysafflor yellow A (HSYA), an active constituent of *Carthamus tinctorius L* reduces atherosclerotic plaque formation in the diabetic mouse model via the inhibition of ACSL4 and HUVEC ferroptosis, and upregulation of GSH-Px, SLC7A11 and GPX4. [110], while Astragaloside IV (AS-IV), the primary constituent of *Astragalus* membranaceus, attenuates atherosclerosis and hepatic steatosis by inhibiting the phosphorylation of JNK, ERK1/2, p38 and NF-κB, and downregulating iNOS, VCAM-1and IL-6 [111]. Tanshinone IIA (Tan IIA), one of the main constituents of S*alvia miltiorrhiza Bunge,* attenuates atherogenesis by inhibiting oxLDL-induced NLRP3 inflammasome activation and downregulating NF-κB-driven pro-inflammatory cytokine accumulation [112].

Overall, there are convincing evidence suggesting the role of ferroptosis in cardiovascular events and atherosclerosis; hence, reduction of ferroptotic stress may be a new therapeutic strategy for atherogenicity and vascular smooth muscle cell phenotype alteration [113–116]. Additionally, an improved understanding of the multifaceted interactions of iron metabolism, lipid peroxidation, and antioxidants during ferroptosis provides some potential therapeutic targets for cardiovascular disease and atherosclerosis [116].

### Neurodegeneration and stroke

**Fig. 3:**
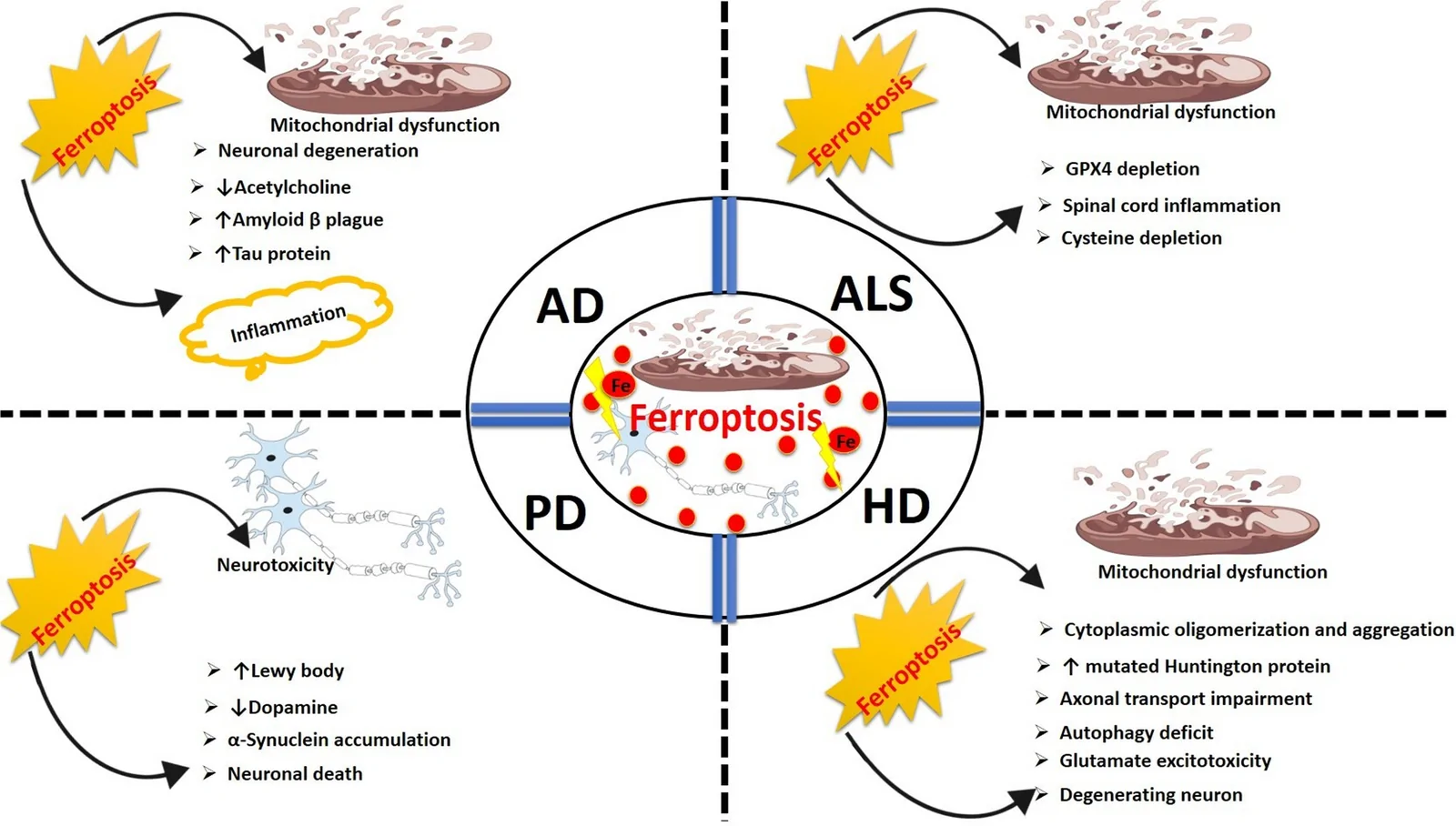
The role of ferroptosis in the pathogenesis of neurodegenerating diseases. Ferroptosis plays a crucial role in the development of neurodegenerating diseases, especially Alzheimer’s disease (AD), amyotrophic lateral sclerosis (ALS), Parkinson’s disease (PD), and Huntington’s disease (HD). Ferroptosis induces mitochondrial dysfunction and upregulation of pro-inflammatory cytokines, which in turn promotes inflammation. In AD, this is associated with increased Tau protein, amyloid β plague accumulation, neuronal dysfunction, and reduced acetylcholine. In ALS, ferroptosis is accompanied by glutathione peroxidase 4 (GPX4) depletion, spinal cord inflammation, and cysteine depletion. In PD, there is increased Lewy body and α-Synuclein accumulation, reduced dopamine, and neuronal death. In HD, ferroptosis leads to cytoplasmic oligomerization and aggregation, increased mutated Huntington protein, impaired axonal transport, autophagy deficit, glutamate excitotoxicity, and degenerating neurons

ROS generation and lipid peroxidation are the main events that trigger iron-dependent non-apoptotic cell death and result in neurodegenerative diseases and stroke. Iron deposits in some regions of the brain oxidize and lead to neuronal damage, which is associated with neurodegenerative disorders, such as amyotrophic lateral sclerosis (ALS), Alzheimer’s disease (AD), Huntington’s disease (HD), and Parkinson's disease (PD) (Fig. 3). Ashraf et al. [117] reported excessive iron in the neurodegenerative mechanism during aging and iron dysregulation leading to lipid peroxidation and cell death in AD. In a related context, Do et al. [118] demonstrated that aberrant iron accumulations in the substantia nigra are responsible for ferroptosis, which leads to neuronal death that typifies PD. These observations highlight ferroptosis as a mechanistic link between iron dysregulation and neuronal death in the course of neurodegeneration. Several studies have also reported the role of ferroptosis in stroke pathophysiology. In ischemic stroke, oxygen and glucose deprivation in the brain leads to ROS and lipid peroxidation that promote ferroptotic cell death [119, 120]. Hemorrhagic stroke due to ferroptosis from iron released from circulating red blood cells. In hemorrhagic stroke, the red cells in the hematoma lyses, releasing haemoglobin that in turn breaks down into heme and iron, leading to iron accumulation. The accumulated iron participates in Fenton reaction, generating highly toxic hydroxyl radicals that leads to ROS production, depletion of antioxidant enzymes, such as GSH and GPX4, and ferroptosis [121].

In a ferroptotic cascade, the dysfunction of iron homeostasis is followed by oxidative degeneration [122, 123]. Weiland et al. [124] linked ferroptotic cell death to neurodegeneration. Ferroptosis causes distortion of the microenvironment of the brain and neuronal cell death in stroke and neurodegenerative diseases by modulating the Nrf2/p53/STAT3 pathway that regulates ferroptotic vulnerability in the central nervous system. Moreso, ferroptosis-related regulators, especially GPX4 and system xc⁻ (SLC7A11), are closely linked to glioma patient survival, and the induction of ferroptosis may prevent resistance to chemotherapy drugs like temozolomide by modulating ferroptosis-related non-coding RNAs (ncRNAs), such as miRNAs, lncRNAs, and circRNAs [125].

### Cancer

Ferroptosis plays a major role in several cancer types, such as lung, breast, and liver, because it represents a distinct mode of regulated cell death driven by iron-dependent lipid peroxidation. Its significance in cancer arises from several key mechanisms. First, several cancer cells exhibit altered iron metabolism and exaggerated oxidative stress [126], making them particularly susceptible to ferroptosis induction. Typically, malignant cells demonstrate iron dysmetabolism, such as overexpression of transferrin receptor 1 (TFR1) [127] that increases iron uptake and promotes oxidative damage. Also, ferroptosis inducers can selectively eliminate malignant cells that are resistant to apoptosis [128], providing promising therapeutic strategies. Moreover, malignant cells often undergo metabolic reprogramming that affects oxidative stress pathways, which can be exploited to trigger ferroptosis selectively [129]. Cancer cells frequently undergo metabolic reprogramming, shifting to aerobic glycolysis, glutaminolysis, and altered lipid synthesis, to prioritize rapid proliferation, biomass production, and survival in harsh tumor microenvironments [130]. This leads to dysregulation of oxidative stress signaling, increasing ROS generation and upregulating incomplete antioxidant defenses like GPX4. This implies selective ferroptosis in cancer cells, sparing normal cells with a balanced redox state, and can be exploited in cancer treatment.

Thyroid cancer, like other malignancies, demonstrates abnormal iron metabolism and oxidative stress pathways, making it vulnerable to ferroptosis-based interventions. A recent study indicates ferroptosis plays a role in thyroid cancer cell death [131, 132], especially given the high oxidative environment of thyroid tissue due to its hormone synthesis function. Thus, targeting ferroptosis may provide novel therapeutic strategies for thyroid cancer. Thyroid cancer (TC), the most common thyroid disease, is the most prevalent endocrine cancer and is growing rapidly around the world [133]. TCs are histologically classified into four main classes: follicular thyroid carcinoma (FTC), papillary thyroid carcinoma (PTC), anaplastic thyroid cancer (ATC), and medullary thyroid cancer (MTC) based on the nature and extent of tumor differentiation [134, 135]. PTC is the most common pathology, with a relatively favorable prognosis; however, ATC is rare and clinically lethal [136, 137]. TC is very heterogeneous across and within tumors, as can be observed from histopathology, genetics, and epigenetic modifications [138, 139]. Notably, the immune microenvironment plays a major role in the spread and metastasis of TC [140]. Other variables include thyroid hormones, iodine, and genetics, which influence TC progression and pathogenesis by regulating cellular growth, differentiation, and metabolism within the tumor microenvironment [141–144].

Polymorphisms in genes coding for ferroptosis are associated with TC risk [145]. APOE-rs429358, BCL3-rs34698726, and rs8100239 were also implicated in TC risk [146]. That is, ALKBH5, a tumor suppressor that is very lowly expressed in tumors, functions as a tumor suppressor with the help of m6A [147, 148]. ALKBH5 was decreased in both TC tissues and cells [149]. Biological behavioral studies indicated that ALKBH5 overexpression led to increases in Fe2 + and ROS, down-regulation of GPX4 and SLC7A11 expression, and hence TC cell proliferation [146]. Furthermore, both in vivo and ex vivo studies confirmed that ALKBH5 inhibited TC progression by modulating TIAM1's m6A level, indicating the main mechanism of ferroptosis induced by the ALKBH5-TIAM1-Nrf2/HO-1 axis [150]. Thus, ALKBH5 will be an option for the diagnosis and therapy of TC [145]. In addition, overexpression of *DPP4, TF, MIOX, PGD,* and *TFRC* genes in the prognostic model also worsens ferroptosis, and upregulation of *GPX4, GSS,* and *ISCU* genes suppresses ferroptosis [146]. Yin et al.[146] also identified *GPX4, GSS, ISCU, and DPP4* genes upregulated in PTC tumor tissues, while *MIOX, TFRC*, *TF,* and *PGD* genes were downregulated.

Specifically, epigenetic regulators such as m6A [151, 152], ncRNAs [153], and regulatory proteins such as GPX4 [154], CD71 [155], ETV4 [156], and SIRT6 [157] hinder TC by controlling tumor cell ferroptosis. TC-associated risk prediction models can be built from ferroptosis-associated mRNAs and lncRNAs [146]. To summarise, clinical interventional therapies targeting ferroptosis can open up new diagnostic and therapeutic opportunities in TC management [145, 158].

### Infectious diseases

Moreover, ferroptosis has also drawn much interest in its dual role in infectious diseases. Host defenses and parasite interactions are complex, so it is important to understand how they function in different infections.

Ferroptosis is induced by disturbances in iron metabolism and antioxidant defenses, mainly through glutathione depletion and the loss of glutathione peroxidase 4 (GPX4) [39]. This leads to the accumulation of lipid peroxides, resulting in membrane damage and cell death, both of which may be harmful or beneficial, depending on the context [159]. In bacterial infections, ferroptosis has been linked to different outcomes based on the microorganism involved.

The survival and replication of *Mycobacterium tuberculosis* (Mtb) depend on the induction of ferroptosis in macrophages,which hampers the host's ability to clear the infection [159]. In addition, ferroptosis has been revealed to be an adjustable process to increase the survival of macrophages and expedite the clearance of intracellular bacteria; hence, targeting ferroptosis regulation might hold a potential therapeutic promise [160]. On the other hand, ferroptosis could be a mechanism of defense against ROS overload, detrimental to some bacteria. Iron overloading in bacterial infections may increase the extent of tissue damage through ferroptosis and worsen the outcome of infections [161].

Ferroptosis is also involved in viral infections. The Hepatitis C virus manipulates iron metabolism in the host, increasing cellular levels of iron and subsequently oxidative stress, which triggers ferroptosis and damages the liver [162]. Similarly, human immunodeficiency virus (HIV) has been indicated to cause disturbances in glutathione metabolism, thus leading to ferroptotic cell death [163]. Influenza A also induces ferroptosis in epithelial cells and contributes to lung injury and exacerbates disease severity [164]. The above findings demonstrate the complexity of ferroptosis in viral pathogenesis and its ability to suppress replication or cause tissue pathology.

Plasmodium infection in malaria results in oxidative stress of the erythrocytes, possibly leading to ferroptosis-like cell death, which allows parasites to egress and spread [165]. In addition, fungal pathogens such as *Aspergillus fumigatus* could induce ferroptosis in host cells, therefore facilitating infections [166]. These interactions demonstrates that ferroptosis is an integral part of the life cycle of several different pathogens and affects host tissue integrity.

The immunological implications of ferroptosis are complex because ferroptotic cells produce damage-associated molecular patterns (DAMPs) that can then act as pathogen-associated molecular patterns (PAMPs) to trigger innate immunity. This form of immunogenic cell death can support pathogen clearance; however, excessive ferroptosis will cause inflammation and tissue damage. Ferroptosis-induced inflammation is also associated with sepsis, where lipid peroxidation and iron dysregulation can cause tissue damage and increased mortality [167–169]. In this regard, a balance between host defense and immunopathology requires optimal regulation of ferroptosis.

### Liver disease

Ferroptosis is also essential in the pathogenesis of hepatic diseases. The liver is key in iron and lipid metabolism; hence, it is susceptible to this process. Non-alcoholic Fatty Liver Disease (NAFLD), a spectrum of conditions that range from simple fatty liver (steatosis) to non-alcoholic steatohepatitis (NASH), a more severe form, is characterized by hepatic inflammation and hepatocyte damage [170]. NAFLD is usually accompanied by increased hepatic iron concentration, which promotes the Fenton reaction [171] that results in increased ROS generation and consequent lipid peroxidation. Since the hepatic tissue of NAFLD patients has high levels of lipids, especially PUFAs that are particularly vulnerable to lipid peroxidation, the increased hepatic iron levels and PUFAs trigger ferroptosis and hepatocyte death, promoting inflammation and fibrosis, which are characteristic of NASH [172, 173].

Ferroptosis has been implicated in Alcoholic Liver Disease (ALD). The primary cause of ALD is chronic and excessive alcohol consumption, which, when metabolized, is accompanied by oxidative stress and iron accumulation [174]. The increased hepatic iron accumulation in ALD is due to enhanced intestinal iron absorption and impaired iron transport mechanisms within the hepatic cells [175]. This leads to hepatic inflammation and fibrosis. Furthermore, in Drug-induced Liver Injury (DILI), glutathione is depleted, causing inactivation of GPX4 [176]. The suppression of this antioxidant mechanism promotes lipid peroxidation, which is accompanied by ferroptosis and hepatic cell death. However, the role of ferroptosis in hepatocellular carcinoma (HCC) is complex and double-edged. Many cancer cells, including those in HCC, have developed mechanisms to suppress ferroptosis, allowing them to survive and proliferate [177]. The inhibition of ferroptosis helps cancer cells evade programmed cell death [178]. This pathway has been explored as a promising therapeutic strategy.

### Hematological disorders

The role of ferroptosis in hematological disorders is also complex and two-edged. While it promotes anemia, it may be beneficial in the management of hematological malignancies. Ferroptosis has been reported to play a role in non-malignant hematological disorders, especially those related to red blood cell production and function. In aplastic anemia, iron overload induces ferroptosis in hematopoietic stem cells, resulting in impaired ability to generate new blood cells [179]. Moreover, ineffective red cell production has been associated with ferroptosis in β-thalassemia. Increased iron concentrations from chronic blood transfusions cause iron overload [180], which promotes ferroptosis in red blood cell precursors, resulting in their premature destruction.

On the other hand, ferroptosis has been explored in the management of hematological malignancies, such as leukemia and lymphoma. Just like in hepatocellular carcinoma and other malignancies, leukemia cells often have impaired iron metabolism and are highly susceptible to ferroptosis [181]. However, these malignant cells can evade ferroptosis by upregulating the antioxidant defense system, such as increasing the activity of GPX4 [182] or altering lipid metabolism to reduce the availability of PUFAs, the primary targets of peroxidation [183, 184].

## Ferroptosis and reproductive disorders

**Fig. 4:**
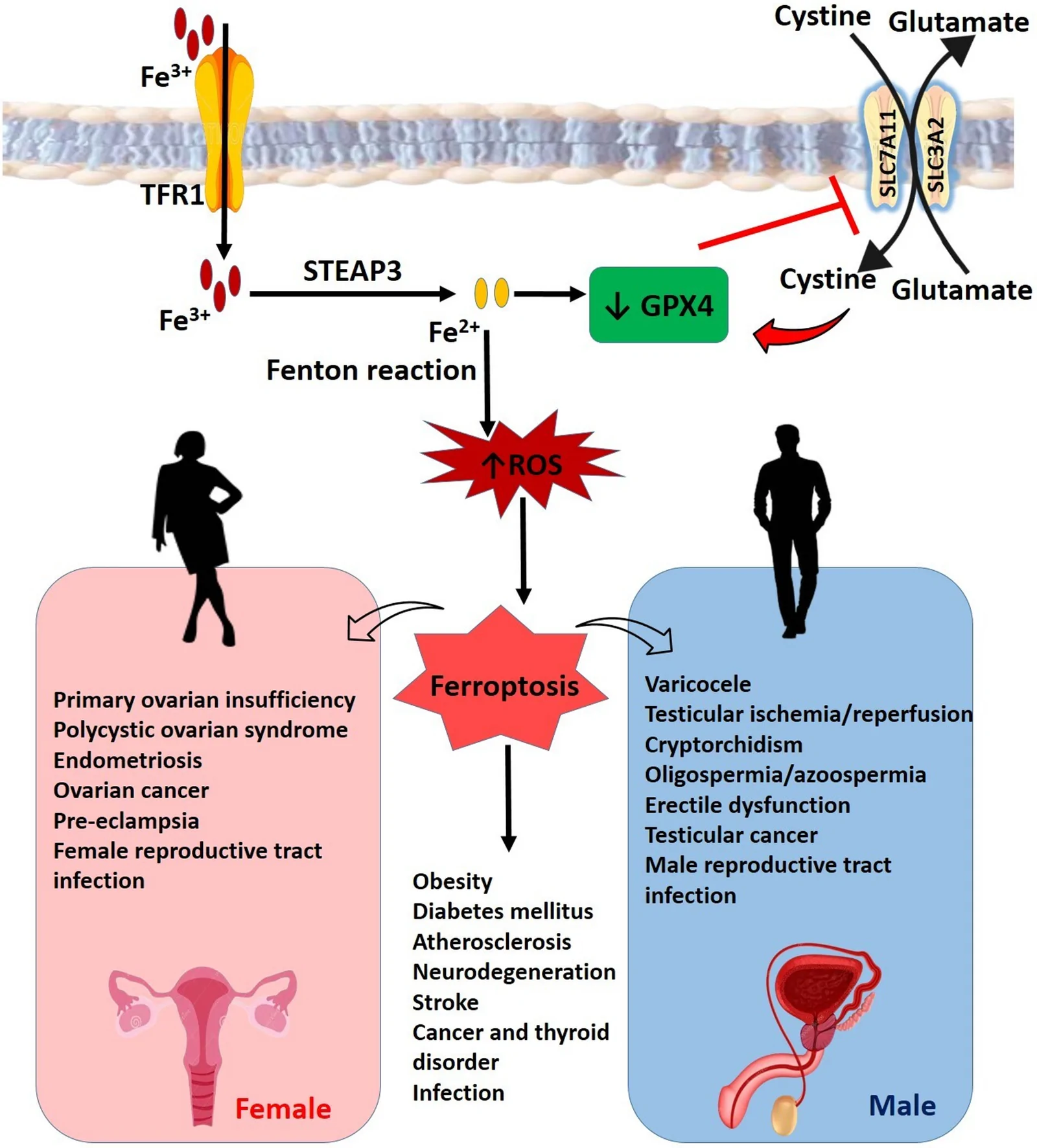
Reproductive disorders associated with ferroptosis. Ferroptosis promotes the development of primary ovarian insufficiency, polycystic ovarian syndrome, endometriosis, ovarian cancer, pre-eclampsia, and reproductive tract infection in females, while in males, it contributes to the development of varicocele, testicular ischemia/reperfusion injury, cryptorchidism, oligospermia, azoospermia, erectile dysfunction, testicular cancer, and reproductive tract infection. More so, ferroptosis plays a role in obesity, diabetes mellitus, atherosclerosis, neurodegeneration, and stroke

There is a rapidly growing decline in human fertility globally, with infertility impacting about 15% of couples; yet the molecular mechanisms remain poorly understood and therapeutic options are limited. Reproductive tissues, especially the testes and ovaries, are highly susceptible to ferroptosis due to their high iron demands, active lipid metabolism, and elevated oxidative stress during gametogenesis. Also, reproductive tract infections exploit ferroptosis to evade host defenses. Thus, a detailed, disorder-specific exploration is essential to identify common therapeutic targets, distinguish pathway-specific interventions, and integrate diverse evidence into a cohesive framework (Fig. 4).

### Male reproductive disorders

#### Varicocele

It has been demonstrated that GPX4 was abundant in normal testis and spermatozoa, and 30% of infertile men with oligoasthenozoospermia expressed GPX4 less frequently in their spermatozoa [185]. Moreover, GPX4 depletion in spermatocytes might lead to irreversible sperm abnormalities, and GPX4 may be involved in spermatogenesis [186]. Notably, heat shock protein 90 (HSP 90), Ca2 + -dependent phospholipase A2 beta (iPLA2), receptor-interacting serine/threonine kinase 3 (RIPK3), receptor-interacting serine/threonine kinase 1 (RIPK1), and gastrin E (GSDME) were associated with ferroptosis, pyroptosis, and necroptosis [187–191]. These results applied only to animal models, but Sun et al. [192] proposed that some of these changes could be observed in human sperm cells, especially in patients with bilateral varicocele.

Bilateral varicocele is a predisposing factor for male infertility [192], with as many as 50% of men with varicocele developing bilateral varicoceles [193]. A varicocele is a pathological venous dilatation and tortuosity of the pampiniform plexus in the scrotum [194]. Varicoceles are found in 15% of all males, 35% in primary infertility, 80% in secondary infertility [195–197] and multifactorial aetiology [194]. Though bilateral varicocele can significantly reduce semen quality, some studies report that varicocele grade and anatomical side influence semen quality [198–200]. Bilateral varicocele not only impairs semen quality, but it also lowers membrane integrity, sperm acrosome integrity, and MMP, and increases ROS, iron level, and apoptosis [192].

In patients with bilateral varicocele, GSH decreases, while ROS generation and iron accumulation increase [192].

#### Testicular ischemia/reperfusion

Testicular ischemia–reperfusion (I/R) is a deviation from physiology resulting from testicular torsion (TT) and detorsion. TT is often regarded as a urologic emergency involving blood restoration following initial acute ischemia [201–203]. The sudden twisting of the spermatic cord causes significant damage to the metabolically active testicular tissue and, ultimately, infertility or subfertility [204–208]. The paradoxical reoxygenation of the testes due to the restoration of blood supply initiates oxidative stress, inflammatory cascades, and apoptotic events [202]. Testicular ischemia/reperfusion from TT has been reported in newborns, children, and adults [209–211]. Testicular ischemia/reperfusion due to torsion is about 65% in males between the ages of 12 and 18 [209]. In a more recent study, the prevalence of TT is 1 in 4000 men [211].

During the ischemia, the metabolic perturbation and iron dysregulation that ensue prime cells for ferroptosis, while the subsequent reperfusion exacerbates ROS generation and cytokine accumulation, and further inactivates GPX4, and augments iron load, leading to a self-amplifying cycle of ferroptotic tissue injury [212].

The Sertoli cell, a somatic cell within the seminiferous tubules that provides essential support for developing germ cells, is the main cellular target of ferroptosis in testicular I/R. Studies using oxygen–glucose deprivation/reoxygenation (OGD/R) models on TM4 Sertoli cells revealed that I/R stress induces cell death via ferroptosis, which is characterized by a rise in iron and lipid ROS levels, mitochondrial shrinkage, and decreased expression of ferroportin (Fpn) [213]. Testicular I/R causes membrane damage and cell death via depletion of cellular GSH and inactivation of GPX4, a major enzyme that prevents ferroptosis [214]. A study in rat model also demonstrated that testicular I/R induces ferroptosis by inhibiting phosphorylation of Akt [215].

#### Cryptorchidism

Cryptorchidism, or undescended testes, is a common congenital anomaly affecting male fertility [216, 217]. It occurs in about 2%−5% of full-term neonates, and the incidence decreases to about 1%−2% within three months; however, the prevalence is higher in premature neonates (up to 30%) [218]. In most cases, testes spontaneously descend shortly after birth, but in approximately 23% of cases, the testis remains undescended unless surgery is performed [218]. Usually, if the spontaneous descent does not occur within the first six months, it might not occur unaided. Men with bilateral cryptorchidism have a six times higher risk of infertility in comparison with individuals with unilateral cryptorchidism and those without cryptorchidism [219]. Maldevelopment of the testes and an unsuitable environment for testicular development, hyperthermia, and anti-sperm antibodies are the central causes of infertility in men with cryptorchidism. Nonetheless, recent studies have shed light on the likely role of ferroptosis as a key player in cryptorchidism-induced male infertility.

In cryptorchidism, the undescended testes remain warmer and thus prone to oxidative stress [220, 221] and iron buildup [222]. The spermatogenic cells in this environment undergo ferroptosis. Ferroptosis impairs the development of sperm, resulting in reduced sperm number, movement, and morphology [7]. Further, chronic ferroptosis also irreversibly destroys testicular tissue and increases infertility [193].

#### Oligospermia and azoospermia

Ferroptosis has been linked to oligospermia, azoospermia, and asthenozoospermia [223–230]. An experimental study revealed that ferroptosis reduced sperm mobility and sperm concentration, while intraperitoneal Fer-1 inhibition of ferroptosis in Nrf2 knockout mice ameliorates oligospermia [225]. In 45 normozoospermic and 42 asthenozoospermic individuals, a greater expression of SLC7A11 and GPX4 genes was lnked to asthenozoospermia [227]. Lipid peroxidation and oxidative stress, which were measured by reduced glutathione and higher levels of MDA, also increased. In addition, mitochondrial membrane potential (MMP) was also reduced, indicating poor mitochondrial function [227]. Di (2-ethylhexyl) phthalate (DEHP) caused Sertoli cell ferroptosis and reduced antioxidant function and lipid peroxidation [230]. Similarly, it has been demonstrated that low semen quality is due to a high ferroptosis level in male smokers [223]. Ferroptosis produces oxidative stress states leading to a caspase-independent loss of sperm life [229]. TM4 Sertoli cell proliferation and activity were suppressed in real-time in the whole body PM2.5 mouse model, inhibiting spermatogenesis and maturation of sperm cells in a mouse model through cell culture and cell cycle assays in a mouse model. The mechanism of ferroptosis-induced sperm disorders, including oligospermia and azoospermia, has been explained [223, 227, 229]. Ferroptosis ensues in rat testes following degenerative morphological changes in the blood-testis barrier of the Sertoli cells due to oxidative stress and lipid peroxidation, which are indicated by the decrease in glutathione level and increase in the concentration of MDA [227, 229]. The upregulation of ALOX15, downregulation of GPX4 protein expression, and induction of electrophile 4-hydroxynonenal and ferroptosis activators, in oxidative stress conditions, stimulate lipid peroxidation [227]. The increased lipid peroxidation inhibits the glutathione defense network by causing a decrease in the levels of glutathione, glutathione-S-transferase, glutathione peroxidase, catalase, and superoxide dismutase, and the downregulation of the GPX4 gene, as well as the impairment of purine metabolism [223, 225, 229]. This redox imbalance induced DNA damage response and cell cycle arrest. The redox imbalance disrupted the metabolic process that ensures the development of spermatogenic cells and ultimately led to oligospermia and caspase-independent apoptotic cell death. TfRC knockdown blocked MEHP-induced ferroptosis by ameliorating mitochondrial dysfunction and decreasing intracellular levels of Fe^2+^ [229]. Ferroptosis in asthenospermia involves an increase in intracellular Fe^2+^ and decreased SLC7A11, suggesting poor sperm mitochondrial function and impaired sperm function [229]. Round spermatid embryos can be made hypersensitive to ferroptosis through the mechanism of acyl-CoA synthetase long-chain family member 4 (ACSL4), which reshapes membrane lipids along a path that favors lipid peroxidation. Therefore, ferroptosis in sperm disorders is caused by iron overload and lipid peroxidation that lead to metabolic disruption and testicular tissue breakdown (Sertoli cells, seminiferous tubule, and epididymis) and redox imbalance with associated sperm DNA damage and finally apoptotic cell death.

#### Erectile dysfunction

Erectile dysfunction is the persistent failure to achieve penile erection and maintain it for satisfactory sexual intercourse [231, 232]. Erectile dysfunction has been described as having multidimensional causes, including psychological, neurogenic, iatrogenic, endocrine, and vasculogenic roles [233]. The global prevalence of erectile dysfunction has been estimated to be 3–76.5% of the total world population in 2019 [234, 235]. Erectile dysfunction is associated with increasing age, with the highest incidence among men more than 40 years old and existing non-communicable comorbidity [235, 236]. These comorbidities may include diabetes, multiple sclerosis, sleep apnea, COPD, glaucoma, hypogonadism, thyroid disorders, sequelae of priapism, depression, hyperlipidemia, stroke, and prostatic hyperplasia with lower urinary symptoms [236]. It could also occur following exposure to medications such as antidepressants, antihypertensives, antipsychotics, opioids, and recreational drugs [235].

In a rat model of diabetes-induced erectile dysfunction, GPX4, SLC7A11, and ACSL4 were noted to be deregulated in the corpus carvernosal smooth muscle cells [237], demonstrating the role of ferroptosis in erectile dysfunction. Similarly, Feng et al. [238] demonstrated that diabetes-induced erectile dysfunction was associated with ferroptosis and via the downregulation of SLC7A11/GPX4/ACSL4 pathway. Low androgen levels has also been shown to induce ferroptosis of rat penile cavernous endothelial cells by upregulating the expressions of TfR1 and ACSL4 and downregulating the expressions of SLC7A11 and GPX4 [239].

Evidence from existing literature explains the involvement of ferroptosis in erectile dysfunction. The upregulation of transferrin receptor 1 protein, intracellular Fe^2+^, ROS, ACSL4, and malondialdehyde, and downregulation of intracellular GSH, GPX4, SLC7A11, eNOS, phospho-eNOS, nitric oxide, cyclic guanosine monophosphate, Ras homolog gene family member A (RhoA)/Rho-associated protein kinase (ROCK) pathway, and PRDX2 explains the mechanistic pathway implicating ferroptosis in erectile dysfunction [238–242]. Iron overload initiates ROS generation, depletion of cellular antioxidant and oxidative stress, which is associated with the downregulation of *SLC7A11* and *GPX4* suppressor genes, leading to ferroptosis of the corpus carvenosum cells and erectile dysfunction.

#### Testicular cancer

Testicular cancer is a leading cause of male infertility, affecting thousands of men worldwide [243]. While traditional treatments like chemotherapy and radiation have improved survival rates, they are accompanied by devastating costs such as irreversible damage to reproductive health [244–247]. Although there is a paucity of data linking ferroptosis to testicular cancer-induced male infertility, available evidence is convincing.

**Fig. 5:**
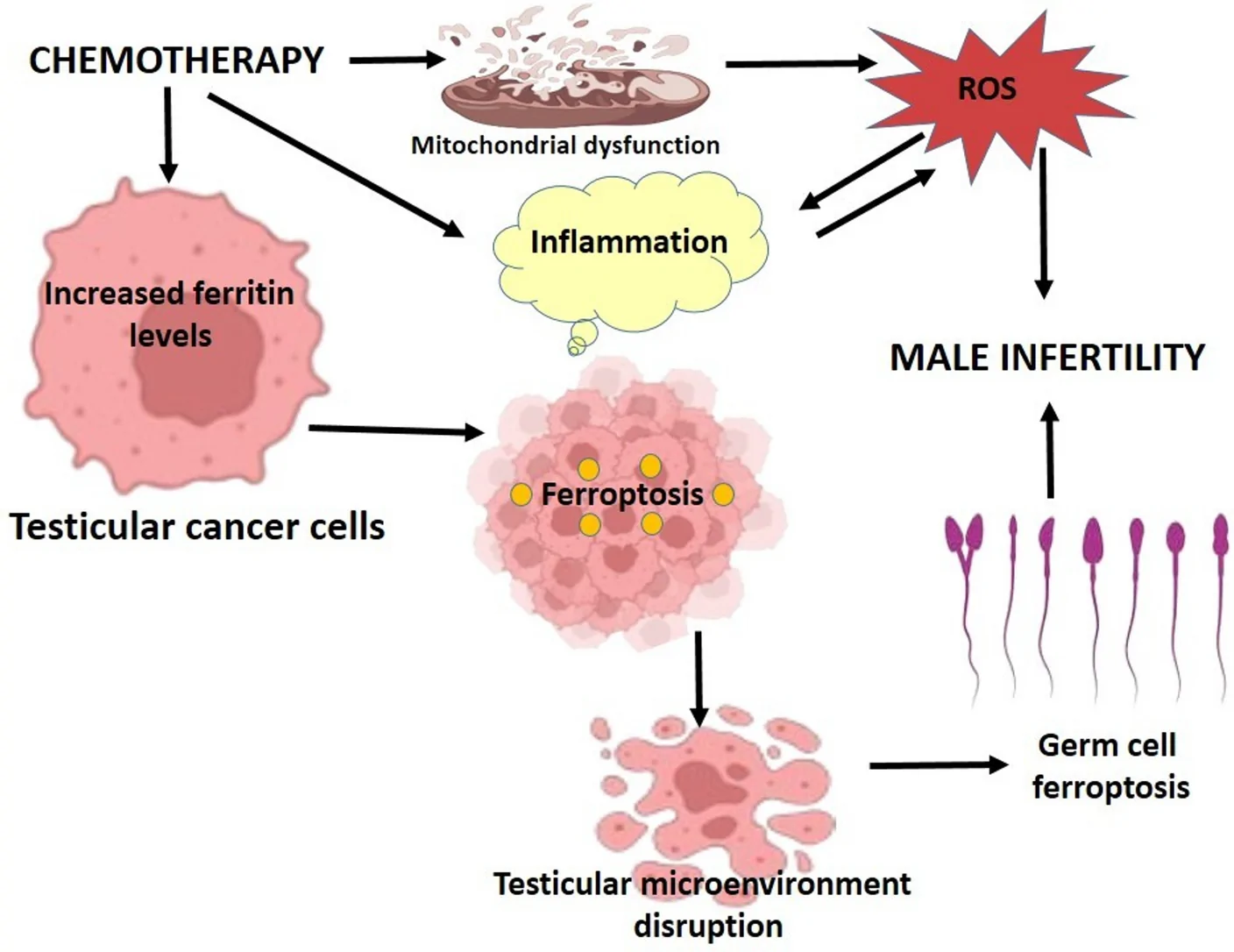
The role of ferroptosis in testicular cancer and its impact on male infertility. Testicular cancer cells show elevated ferritin levels and iron accumulation, contributing to sensitivity to chemotherapy. The increased iron levels and chemotherapy further elevates reactive oxygen species (ROS) and disrupts mitochondrial function, resulting in lipid peroxidation and ferroptosis in both cancer and healthy testicular cells, including spermatogonia. The resulting cell death and inflammation damage the testicular microenvironment, impairing spermatogenesis and causing male infertility

Testicular cancer cells exhibit increased levels of ferritin, a marker of ferroptosis, which contributes to their sensitivity to chemotherapy [248, 249]. However, this process also affects the surrounding healthy cells, including spermatogonia, leading to male infertility. Although chemotherapeutic agents are effective in the management of testicular cancer, they enhance ROS accumulation, which in turn trigger lipid peroxidation and lead to ferroptosis [250, 251] in testicular cells. Elevated iron levels from cancer cells [250] and chemotherapy [252] in testicular tissue exacerbate ferroptosis, further damaging spermatogonia and other germ cells. In addition, chemotherapy disrupts mitochondrial function [253], increasing ROS production and ferroptosis. Chemotherapy may also induce inflammation, which promotes ferroptosis, damaging the testicular microenvironment (Fig. 5).

#### Male reproductive tract infections

Male reproductive tract infections (RTIs) are a public health concern, causing infertility and death. RTIs are classified into three types: sexually transmitted infections (STIs), iatrogenic infections, and endogenous infections. Bacteria, viruses, fungi, and parasites can cause RTIs. Common causal factors are Neisseria gonorrhoeae, Chlamydia trachomatis, Human Papillomavirus (HPV), and Trichomonas vaginalis. These infections can lead to infertility by inducing inflammatory responses and negatively impacting sperm production and maturation.

Ferroptosis has been linked to many reproductive disorders and infections. Studies have reported that ferroptosis aids the pathogenesis of infections. Not many studies have specifically demonstrated ferroptosis in male reproductive infections of microbial origin, but reports from some studies strongly suggest that ferroptosis favors the pathogenesis of microorganisms associated with male reproductive tract infections.

GPX4 protects against bacterial infection by inhibiting ferroptosis. Amaral et al. [254] reported that macrophage cell death in *Mycobacterium tuberculosis* infection was associated with a decline in GSH and GPX4 and free iron accumulation, as well as ROS formation and lipid peroxidation. Although the exact mechanism by which ferroptosis causes reproductive infection has not been fully demonstrated, existing evidence shows that system Xc and GPX4, mitochondrial voltage-dependent anion channel (VDAC)-mediated-ferroptosis, and p53-mediated ferroptosis are involved. System Xc is an abundant amino acid in phospholipid bilayers. It plays a role in the transport of cystine and glutamate in and out of the cell [12]. Cystine is essential for GSH synthesis, which reduces ROS using GPX. Whatever induces the inhibition of this amino acid inhibits the uptake of cystine, leading to decreased GPX activity, accumulation of lipid ROS, and reduced antioxidant capacity. These events result in oxidative damage to the cell and ferroptosis [255, 256]. Also, VDAC is the most predominant protein in the outer membrane of the mitochondria, and it is essential in several cellular activities, including mitochondrial bioenergetics, calcium signaling, and cell death. These membrane channels transport ions and metabolites through cells and are involved in ferroptosis. Erastin binds to VDAC and alters the permeability of the outer mitochondrial membrane, leading to VDAC opening**,** mitochondrial hyperpolarization (increased ΔΨm), and enhanced mitochondrial ROS generation that in turn triggers oxidative stress and ferroptotic cell death [257]. Jiang et al. [255] reported that p53 inhibits the uptake of cystine by directly suppressing SLC7A11, the catalytic subunit of the cystine/glutamate antiporter system xc⁻, leading to reduced intracellular cysteine availability for glutathione (GSH) synthesis and GPX4 inactivation since GSH is an essential cofactor for GPX4-mediated detoxification of lipid peroxides. The inactivation of GPX4 leads to ROS accumulation, oxidative membrane damage, and ferroptosis.

*Chlamydia trachomatis*, the most prevalent bacterial sexually transmitted infection, adopts a chlamydial protease-like activity factor (CPAF) that specifically targets SLC7A11 for glutathione degradation and depletion to induce ferroptosis and facilitate progeny release [258]. Also, the inflammatory process in orchitis represses SLC7A11 and GPX4 while upregulating ACSL4 and LPCAT3, creating a pro-ferroptotic state in peritubular myoid cells [259]. Interestingly, treatment with ferroptosis inhibitors, such as Ferrostatin-1 restores SLC7A11, SLC3A2, and GPX4 expression, demonstrating the role of SLC7A11/GPX4 in male reproductive tract infection. Similarly, chronic prostatitis is associated with downregulation of the system Xc⁻/GPX4 pathway and reduced NRF2/HO-1 signaling, indicating susceptibility to ferroptosis [260].

In addition, the role of VDAC and p53/SLC7A11 in reproductive tract infection has been explored. *Chlamydia trachomatis* regulate ferroptosis via the p53/SLC7A11 pathway. A bioinformatics analysis of Chlamydia trachomatis-infected cells revealed a marked activity of p53 signaling [261]. The p53/SLC7A11 axis is an established canonical ferroptosis regulatory pathway in reproductive tract infections broadly. When p53 is activated, it transcriptionally suppresses SLC7A11, limiting cystine uptake and increasing the vulnerability of cells to ferroptosis [261].

*Escherichia coli* (*E. coli*) is significant in male reproductive infection-induced infertility. *E. coli* negatively modulates ferroportin, causing an overload of iron [262]. *E. coli* increases ferric ion and downregulates ferroportin (FPN1), leading to excess ROS production and lipid peroxidation via the Fenton reaction. The study opines that the incubation of *E. coli* activates the oxidation of hemoglobin and increases *heme*, which induces the expression of *heme oxygenase 1* and ferritin while repressing ferroportin expression. This series of activities leads to an accumulation of intracellular iron, ROS, and ferroptosis. Thus, *Escherichia coli* is capable of inducing cell death via an iron-mediated ferroptosis pathway.

*Neisseria gonorrhoeae,* the most common bacterium in the male reproductive system, which causes inflammation of various parts of the male reproductive system, has also been reported to activate ferroptotic pathway. *N. gonorrhoeae* acquires iron from its human host, a prerequisite for its survival and virulence [263]. The bacterium expresses transferrin-binding proteins, lactoferrin-binding proteins, and siderophore uptake systems to pirate iron from host glycoproteins [263]. This hostile iron acquisition may directly influence the labile iron pool available for Fenton chemistry and lipid peroxidation in infected cells.

### Female reproductive disorders

#### Primary ovarian insufficiency

Primary ovarian insufficiency (POI) or premature ovarian failure [264] occurs when the ovaries fail before age 40, resulting in infertility and hormonal dysfunction [265]. POI affects about 1 percent of women under 40 years old and has genetic, autoimmune, and environmental causes. The condition is more common among women with Turner syndrome and those with a family history of POI [266], and it is associated with complications, such as osteoporosis, cardiovascular disease, and depression [267].

The Nrf2/GPX4 Antioxidant Axis is a key protective pathway that is dysregulated in POI [268]. Downregulation of Nrf2 signaling leads to decreased GPX4 and the cystine/glutamate antiporter XCT (SLC7A11), resulting in impaired glutathione (GSH) synthesis, reduced antioxidant capacity, and subsequent lipid peroxidation. Conversely, activating this pathway with agents like melatonin or salidroside-enhanced mesenchymal stem cells confers protection by restoring redox homeostasis. Also, autophagy-dependent ferroptosis is implicated in POI. The deubiquitinase USP8 promotes ferroptosis by stabilizing Beclin1, a key autophagy regulator, that in turn enhances autophagy, which then the degradation of ferritin (ferritinophagy), releasing free iron and triggering lipid peroxidation and ferroptotic cell death in granulosa cells. More so, specific gene mutations and epigenetic modifications predispose to ferroptosis. BNC1 deficiency induces ferroptosis via the NF2-YAP pathway, contributing to POI in a pedigree model [269]. Furthermore, HDAC3 acts as an essential epigenetic suppressor of ferroptosis in pregranulosa cells; its loss triggers ferroptosis and depletes the primordial follicle reserve [270].

#### Polycystic ovarian syndrome

PCOS, now referred to as polyendocrine metabolic ovarian syndrome (PMOS), is a complex endocrinopathy with associated metabolic dysfunction. It is the most common endocrine condition in women over their reproductive years [271, 272]. The manifestations include polycystic ovarian tissue, insulin resistance, anovulation or infrequent ovulation, hyperandrogenemia, and lowered female fertility [273]. Studies have shown that women with PCOS have significantly increased ROS, leukocytes, and ferritin levels, a key marker of ferroptosis, in ovarian granulosa cells [274, 275]. When compared with obese patients who did not have PCOS, obese women with PCOS have higher levels of serum ferritin [276]. These studies demonstrate that PCOS is associated with increased iron accumulation and ferroptosis. Moreover, hyperinsulinemia, which is accompanied by insulin resistance in PCOS, promotes iron uptake in tissues [276, 277] and prevents iron release from macrophages [278], resulting in the accumulation of iron [279].

Excessive accumulation of iron in the ovarian tissues induces lipid peroxidation and ferroptosis [280], worsens insulin resistance and/or hyperinsulinemia, and increases LH sensitivity, thus inhibiting follicular development [274], ovulatory function, and embryonic implantation [281]. These events are associated with NF-kB-mediated upregulation of miR-93-5p, ROS, and MDA levels, and downregulation of GPX4 levels [282]. An experimental study showed that ferroptosis may mediate PCOS-associated fetal loss via the downregulation of GPX4 and GSH levels and activation of ERK/p38/JNK, and ferroptosis-related genes *TFRC*, *SLC7A11*, and *ACSL4* in the pregnant uterus [283]. Interestingly, ferroptosis inhibitors have been reported to improve PCOS-like symptoms and attenuate uterine and placental dysfunction in animal models of PCOS [284].

#### Endometriosis

Endometriosis is a long-term gynecological disease that manifests as a layer of endometrial tissue lying outside the uterus with associated chronic pelvic pain, dysmenorrhagia, and infertility [285]. It affects about one in ten women in the reproductive age globally. The incidence of endometriosis varies between 2 and 22%, depending on the sample and the diagnostic criteria [286]. Multiparity, the history of the disease, and shorter menstrual cycles are major risk factors for endometriosis [287]. Although the exact cause of endometriosis is yet to be identified, retrograde menstrual flow/periods, genetic predisposition, and immune dysfunction have been implicated [288]. According to the reports of Global Burden of Disease (GBD) in 2021, the global prevalence of endometriosis among women of childbearing age is estimated at 22.28 million cases**,** corresponding to an age-standardized prevalence rate of 1023.8 per 100,000 [289].

Current evidence suggests a possible role of ferroptosis in the development of female infertility in women with endometriosis. Iron overload is one of the major characteristics of endometriosis and is associated with the growth and survival of endometriotic lesions [290, 291]. Elevated iron levels and oxidative stress in endometriotic lesions result in ferroptotic death of endometrial and follicular cells [292]. The death of endometrial and follicular cells induced by ferroptosis can lead to various reproductive dysfunctions [293], such as reduced oocyte quality, endometrial resistance, and uterine contractility, that are contributing factors to infertility in patients with endometriosis [294]. Ferroptosis in the ovarian follicles reduces the number of viable oocytes and their quality, resulting in decreased fertility or infertility [294].

#### Ovarian cancer

Ovarian cancer is a fatal gynecologic disease considered the most lethal female tumor, but often overlooked as it is usually asymptomatic in its early stages and, thus, difficult to manage [295–298]. Hence, over 70 percent of ovarian cancer cases are not diagnosed until they have progressed to stage III or IV [297, 298]. Interestingly, research shows this malignancy does not originate in the ovaries all the time, but from the fallopian tube and other epithelial sites in the pelvis [297]. They metastasize to the ovary from these sites, causing different reproductive disorders. The risk of this cancer increases with age, with most cases reported in perimenopausal and postmenopausal ages [295].

**Fig. 6:**
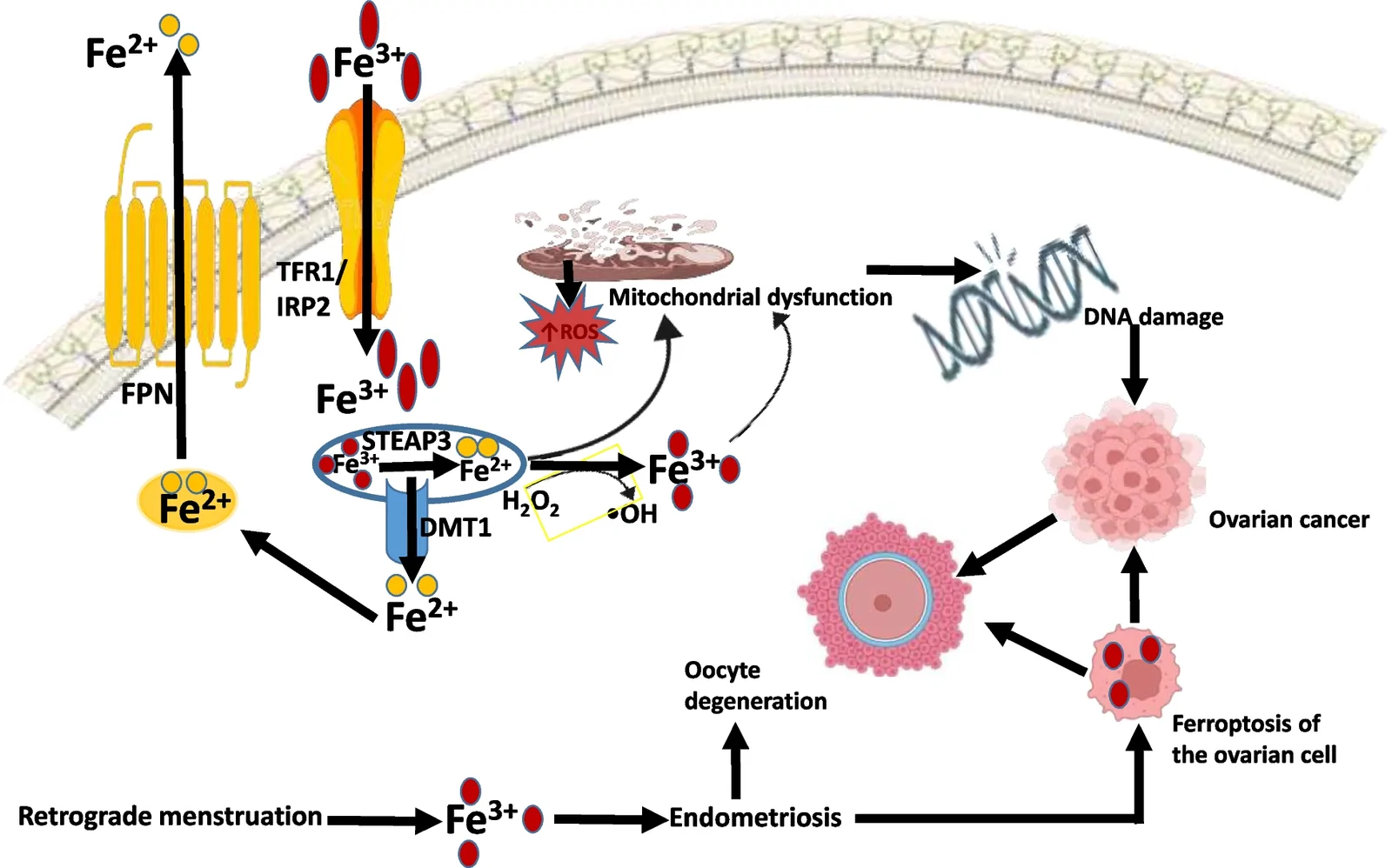
The role of ferroptosis in ovarian cancer development. Overexpression of transferrin receptor 1 (TFR1) and iron regulatory protein 2 (IRP2), alongside decreased ferroportin (FPN), drives intracellular iron accumulation in ovarian cells, causing iron overload. This iron overload promotes oxidative stress through Fenton reactions, resulting in DNA damage contributing to cancer progression. Additionally, retrograde menstruation promotes endometriosis, a risk factor linked to ovarian cancer. Excess iron accumulation in follicular fluid induces ferroptosis in granulosa cells responsible for steroidogenesis and oocyte development, leading to impaired fertility

Compelling evidence demonstrates the involvement of iron in ovarian cancer development. Immunohistochemistry report shows that TFR1 is significantly higher, while the expression of FPN is decreased in high-grade serous ovarian carcinoma (HGSOC) [299]. In addition, there is an upregulation of iron regulatory protein 2 (IRP2) expression in the ovarian tissue in ovarian cancer [300], which further indicates iron overload in the ovarian cells. Iron overload induces oxidative stress via Fenton reactions, and thus, mutagenesis is initiated in the face of dwindling antioxidant defense [301, 302]. In endometriosis, a risk factor for ovarian cancer, oxidative stress results in DNA damage and retrograde menstruation [303, 304]; this may explain the link between both reproductive disorders (Fig. 6).

#### Pre-eclampsia

Pre-eclampsia is a multisystemic disease of hypertension (systolic blood pressure > 140 mm Hg and diastolic at 90 mm Hg measured two times at least 4–6 h apart) in the presence of proteinuria (0.3 mg protein in 24-h urine test or 1 + using dipstick) without edema and first appearing after the 20th week of gestation in a normotensive patient and disappearing completely by 42 days post-partum. [305]. It also involves maternal multiple organ damage, such as neurological, renal, and hepatic dysfunctions, as well as uteroplacental dysfunction [306], which may lead to fetal growth restriction or stillbirth.

Altered iron homeostasis and decreased iron metabolism-related protein expression, such as FTH1, FTL, and FPN1, key regulators of ferroptosis, have been observed in pre-eclamptic placentas [307]. This may promote the Fenton reaction and enhance ROS generation, leading to lipid peroxidation and ferroptosis. In pre-eclamptic patients, the presence of high concentrations of lipid peroxidation products, such as 4-hydroxynonenal (4-HNE) [308, 309], and low concentrations of glutathione [309], a powerful antiferroptosis antioxidant, in placental tissues could be trigger ferroptosis in placental tissue. Alterations in ferroptosis-associated gene expression, including overexpression of ferroptosis inducers (e.g., TP53) [310] and downregulation of ferroptosis suppressors (e.g., SLC7A11) [311] have been observed in pre-eclamptic placentas. More so, PUFA level is higher in pre-eclamptic placentas than in normotensive women [312], which predisposes pre-eclamptic placenta to oxidative damage in the presence of iron accumulation.

Vasospasm of the small arteries, loss of vascular endothelium, and reduced organ perfusion that are seen in pre-eclampsia lead to hypoxia-reperfusion or lipotoxic injury and ferroptosis [313], trophoblast cell death and loss of placental function [314]. Further, ferroptosis induces placental inflammation and oxidative stress, incomplete trophoblast invasion, and mal-remodeling of the spiral arteries of the uterus, leading to pre-eclampsia [315].

#### Female reproductive tract infections

Reproductive tract infections (RTIs) are prevalent among women of reproductive age and are associated with pelvic inflammatory disease, preterm delivery, and infertility. Lactobacilli help to create vaginal homeostasis [316]; depletion of this bacteria causes bacterial vaginosis (BV) and may impair fertility. Just as in male reproductive tract infection, ferroptosis plays a role in the pathogenesis of female reproductive tract infections primarily via the disruption of the System Xc⁻–GSH–GPX4 antioxidant axis and iron dysregulation.

Ferroptosis is a major driver of tissue damage in endometritis, an inflammatory condition of the endometrium that is usually triggered by bacterial infection. Studies using lipopolysaccharide (LPS)-induced and *Staphylococcus aureus*-induced endometritis models in mice have confirmed the occurrence of ferroptosis, characterized by a rise in MDA and iron levels as well as a decline in GPX4 and GSH [317, 318]. The activation of the Farnesoid X receptor (FXR) and the Aryl hydrocarbon receptor (AhR) has been shown to protect against endometritis by inhibiting ferroptosis [317, 319], suggestive of its role in the pathogenesis of endometritis.

Also, ferroptosis may play a major role in the pathogenesis of Human Papillomavirus (HPV) infection, promoting cervical carcinogenesis. Ferroptosis is active during early precancerous stages (squamous intraepithelial lesions), but as the disease progresses to cervical squamous cell carcinoma, cancer cells develop resistance to ferroptosis [320]. This resistance is mediated through c-Myc/miR-142-5p/HOXA5/SLC7A11 pathway, which suppresses lipid peroxidation and iron accumulation [321].

However, ferroptosis plays a dual role in vaginal candidiasis. *Candida albicans* infection induces ferroptosis in vaginal tissues and immune cells, which paradoxically worsens the disease. *C. albicans* infection triggers ferroptosis in macrophages, key immune cells responsible for fungal clearance [322], leading to macrophage ferroptotic death that is characterized by lipid ROS accumulation, Fe^2^⁺ overload, GSH depletion, and downregulation of GPX4 and SLC7A11 [322]. Macrophage ferroptosis impairs antifungal activity, contributing to chronic inflammation and tissue damage; however, treatment with the ferroptosis inhibitor ferrostatin-1 (Fer-1) reduces fungal burden, inflammation, and vaginal pathology, while restoring macrophage viability and antifungal capacity [322]. Conversely, a recent study found that exposure of *C. albicans* to ferrous sulphate (FeSO₄) triggers classic ferroptosis in the fungus, characterized by classical features like Fe^2^⁺ overload, ROS eruption, and lipid peroxidation [323]. This strategy provides a potential alternative to conventional antifungals, addressing the growing problem of drug resistance.

Ferroptosis plays a dual, stage-dependent role in *Chlamydia trachomatis* infection. The bacterium suppresses ferroptosis in the early phase of infection to protect its replication niche and induces it late via CPAF-mediated SLC7A11 degradation to facilitate progeny release. CPAF proteolytically cleaves SLC7A11 within the host cell, eliminating cystine uptake capacity and suppressing GPX4 activation [258].

## Therapeutic translation: harnessing and halting ferroptosis

### Biomarkers for ferroptosis detection and monitoring

Detection and monitoring of ferroptosis is essential to the pathology and treatment of associated diseases. Several biomarkers have been identified for the detection and monitoring of ferroptosis. An important marker for ferroptosis is the overload of intracellular ferrous iron (Fe2 +). A high level of Fe2 + is detectable by biochemical tests, Prussian blue staining, or probes such as FerroFarRed [324]. Higher levels of the transferrin receptor (TFRC), which mediates cellular iron transport, are another good indicator [125].

Another hallmark of ferroptosis is lipid peroxidation (or oxidation of PUFAs), which produces lipid hydroperoxides. The hydroperoxides can be detected by HPLC or mass spectrometry [325]. A rise or decrease in GPX4 activity, an enzyme that prevents lipid peroxidation, has also been shown to be an important biomarker of ferroptosis [326, 327]. Recently, long non-coding RNAs (lncRNAs) have been proposed as markers of ferroptosis [328–330]. The use of serum ACSL4 as a serological marker is also gaining attention [331] as it demonstrate a high predictive value. The use of these biomarkers allows ferroptosis to be characterized during the progression of disease and thus, therapeutic strategies can be promptly instituted.

### Current and emerging therapies

Therapeutic strategies targeting ferroptosis aim to modulate this pathway by blocking or activating cell death (Table 1).

**Table 1:** Inducers of ferroptosis

Molecules	Targets	Mechanisms	References
ACSL4	Fatty acids	Conversion of free fatty acids into fatty CoA ester	[332]
**Inducers**			
As-lncRNA SLC7A11	*SLC7A11*	Inhibition of System Xc^−^	[333]
BAP1	*SLC7A11*	Inhibition of System Xc^−^	[334]
BECN1	*SLC7A11*	Inhibition of System Xc^−^	[335]
DPP4	NOXs	Lipid peroxidation	[336]
EGFR	MAPK	Cystine deprivation	[337]
Erastin	*SLC3A2*	Inhibition of System Xc^−^	[335]
FANCD2	GPX4 Iron metabolism genes	GPX4 inhibition and cellular iron availability	[337]
Glutamate	*SLC3A2*	Inhibition of System Xc^−^	[335]
HO-1	Heme	Heme degradation	[338]
HSPB1	Actin dynamics	Cellular iron availability	[15]
IFNγ	*SLC7A11 SLC3A2*	Induction of lipid peroxidation	[339]
miR-155	ROS	Upregulation of ROS levels via inhibition of FOXO3a expression	[340]
miR-206	ROS	Upregulation of ROS levels via blockade of SOD1	[341]
NCOA4	Iron and ROS	Promotion of iron accumulation and ROS generation	[14]
p53	*SLC7A11*	Inhibition of System Xc^−^	[342]
p53RRA lncRNA	G3BP1	p53 nucleus retention	[343]
SAT1	Lipoxygenases	Lipid peroxidation	[344]
SLC40A1	Iron metabolism	Upregulation of FPN activity	[335]
Sorafenib	*SLC3A2*	Inhibition of System Xc^−^	[335]
sulfasalazine	*SLC3A2*	Inhibition of System Xc^−^	[335]
**Inhibitors**			
FSP1/AIFM2	Mitochondrial apoptosis	Inhibition of ferroptosis via an apoptosis-independent mechanism	[335]
HSPB1	TFR1	Inhibition of TFR1 expression and cellular iron uptake	[15]
IREB2	FTL and FTH1	Up-regulation of FTL and FTH1 and blockade of erastin-induced ferroptosis	[16]
miR-137	*SLC1A5*	Accumulation of MDA	[345]
miR-25	ROS	Suppression of ROS generation by targeting NOX4	[346]
miR-448-3p	ROS	Inhibition of NOX2-dependent ROS generation	[347]
Nrf2	*SLC7A11, HO-1, GPX4, G6PD*	Inhibition of System Xc^−^, cellular iron availability	[40]
p53	P21	Boosts antioxidant defense	[348]

This table summarizes key pharmacological agents that modulate ferroptosis, categorized by their primary molecular targets and mechanisms of action. Ferroptosis inducers (left columns) promote cell death by inhibiting the System Xc⁻–GSH–GPX4 axis (e.g., erastin, sulfasalazine, sorafenib, glutamate), disrupting iron metabolism (e.g., FINO₂, hemin, NCOA4), or directly enhancing lipid peroxidation via ACSL4 or lipoxygenases. Ferroptosis inhibitors (right columns) suppress cell death through iron chelation (e.g., deferoxamine, deferiprone), direct radical-trapping antioxidant activity (e.g., Ferrostatin-1, Liproxstatin-1, vitamin E), or upregulation of the endogenous antioxidant defense system (e.g., Nrf2 activators). Agents are grouped based on primary targets described in the literature, although some compounds exhibit pleiotropic effects. Abbreviations: *ACSL4* acyl-CoA synthetase long-chain family member 4, *BECN1* beclin 1, *DPP4* dipeptidyl peptidase-4, *FPN* ferroportin, *GPX4* glutathione peroxidase 4, *GSH* glutathione, *HO-1* heme oxygenase 1, *NCOA4* nuclear receptor coactivator 4, *Nrf2* nuclear factor erythroid 2-related factor 2, *PUFA* polyunsaturated fatty acid, *ROS* reactive oxygen species, *SLC7A11* solute carrier family 7 member 11, *TFRC* transferrin receptor

Several small molecules have been demonstrated to be inducers of ferroptosis. Erastin blocks the cystine/glutamate antiporter system Xc and thus depletes glutathione, leading to lipid peroxidation, which leads to ferroptosis in cancer cells [12]. In the same manner, RSL3 directly binds to glutathione peroxidase 4 (GPX4), a proteolytic enzyme responsible for lipid peroxidation and hence ferroptosis [11]. These agents have been shown to cause ferroptosis in various cell lines, indicating potential as therapeutic agents.

Some pharmacological inhibitors of ferroptosis have been identified that inhibit this process by modifying regulatory enzymes and iron metabolism. Both ferrostatin-1 and liproxstatin-1 are shown as inhibitors of lipid peroxidation and ferroptosis across a range of cell types. These agents act by eliminating reactive oxygen species (ROS) or directly affecting iron metabolism enzymes and therefore reduce iron-related oxidative stress [12]. Deferoxamine is an iron chelator frequently used to prevent ferroptosis by making iron less bioavailable and hence less likely to generate iron-induced ROS. Recent research showed that deferoxamine could also be used as an adjuvant therapy for neurodegenerative disorders such as Alzheimer's, in which ferroptosis is a key pathological mechanism in neuronal damage [349].

Natural compounds have been looked to for their ferroptosis-inducing properties, too. For example, an antimalarial agent called artesunate causes ferroptosis in some cancer cells by iron-dependent mechanisms [350]. Moreover, sulfasalazine, commonly used in chronic inflammatory conditions, blocks system Xc, promoting ferroptosis in glioma cells [38]. Besides drugs made from chemicals, some plant-based products have also been shown to modulate ferroptosis (most commonly via antioxidant and anti-inflammatory agents).

The active ingredient in turmeric is curcumin, which is known to defend cells against ferroptotic cell death. It inhibits ROS generation and increases antioxidant enzyme activity to prevent lipid peroxidation [114]. Curcumin prevents malignant growth and neurodegeneration by inhibiting ferroptosis [114]. Another plant-derived compound from milk thistle called silymarin [351] has also been reported as being anti-ferroptotic in liver cells by lowering oxidative stress and lipid peroxidation [307]. Silymarin also modulates Kim-1/Nrf2/HO-1 to prevent renal amyloidosis and ferroptosis [327]. Thus, silymarin is a promising candidate in the management of ferroptosis.

Ferroptosis involves a loss of cellular antioxidants, further aggravating oxidative stress. Therapeutic approaches to augment the antioxidant response in the body could prevent ferroptotic injury. N-acetylcysteine (NAC), a precursor of glutathione, has been studied as a mechanism for re-energizing cellular antioxidants and preventing ferroptosis. NAC also replenishes glutathione, a vital factor that can neutralize ROS caused by iron-induced cellular damage [352]. *Zingiber officinale*Roscoe (Ginger) (Zingiberaceae)and its phytochemical constituents, 6-gingerol and zingerone, have been demonstrated to inhibit ferroptosis during chemotherapy to prevent injuries to non-target organs. These nutraceuticals upregulate antioxidants, including GPX4, prevent iron accumulation, and modulate Bax/Bcl-2, caspases‐3 and − 9, ERK1/2/Nrf2/p65/NF‐κB, p38/MAPK, p53, TNF‐α, ROS/NF‐κB/COX‐2, and SAPK/JNK signaling pathways [353]. On the other hand, increased expression of certain proteins, such as ferroportin, an exporter of iron, may also regulate intracellular iron and decrease the incidence of ferroptosis [15].

Nanotechnology also offers novel approaches for the modulation of ferroptosis. Because of their unique physicochemical features, nanomaterials show promising new roles in the treatment of cancerous cells [354, 355]. For example, nanoparticles of iron oxide increase intracellular levels of iron, thus promoting ferroptosis [356]. In addition, nanocarriers can selectively deliver ferroptosis-inducing molecules directly to the tumor cells, which thus decreases systemic toxicity and increases the potency of treatment [128].

Another promising approach involves the tumor microenvironment. Acidity and hypoxia stimulate the ferroptosis of tumors, and manipulation of these environmental factors triggers cancer cell death [357, 358]. In addition, several combination regimens incorporating ferroptosis inducers with immune checkpoint inhibitors showed potential synergy in preclinical models and improved anti-tumor immunity [359].

Novel gene therapy strategies could represent a potentially effective approach to control ferroptosis. To this end, small interfering RNA (siRNA) and CRISPR/Cas9 methodologies are currently being investigated to target and silence genes associated with ferroptosis, including acyl-CoA synthetase long-chain family member 4 (ACSL4) and transferrin receptor (TFRC) [336, 360, 361]. The aforementioned genetic manipulations may subsequently be utilized to selectively induce or inhibit ferroptosis, thereby offering significant therapeutic potential forthetreatmentofcancer andneurodegenerative diseases. For example, the knockdown of ACSL4 downregulates ferroptosis in certain cancer cells, thus favoring cell survival and proliferation. The regulation of TFRC expression level may alter iron uptake and retention, ultimately impacting ferroptosis [336, 360, 361].

#### Inducers for cancer therapy

Recent research is actively exploring ferroptosis inducers as novel cancer treatment modalities, specifically in cancer resistant to conventional modalities [362]. The key classes of inducers are small molecules, including elastin, RSL3, and sorafenib, which arrest cystine uptake or inhibit glutathione peroxidase 4 (GPX4) to cause lethal lipid peroxidation in cancer cells [363]. Artemisinin derivatives (such as artesunate and dihydroartemisinin) are natural products that show anti-tumor activity in vitro and in vivo and exploit the iron-rich composition of cancer cells to induce ferroptosis and are particularly effective against leukemia, colon, ovarian, and glioma cancer cell lines [364]. Moreover, the preclinical testing of new compounds such as phospholipid degraders and iron oxide nanoparticles is already in progress, and this area focuses on selectively triggering ferroptosis in drug-tolerant metastatic cells or cancerous cells resistant to chemotherapy [365]. The combination techniques, which include coupling ferroptosis inducers with immunotherapy or chemotherapy, demonstrate synergistic effects through improving tumor immunogenicity and overcoming resistance, which have been reported in animal models and early-phase research [366]. Although preclinical research yields encouraging findings, clinical translation remains rudimentary, and it includes an active attempt to maximize dosing, delivery, and patient selection [366].

#### Inhibitors for degenerative/ischemic diseases

Ferroptosis inhibitors are being developed to prevent oxidative tissue and organ damage in neurodegenerative and ischemic injury diseases. Most established inhibitors are iron chelators such as deferoxamine (DFO), deferiprone (DFP), and deferasirox (DFX) that inhibit iron-based catalysis of lipid peroxidation and were effective in stroke, spinal cord injury, and acute kidney injury models [367]. Novel oral chelators (CN128 and DFA1) showed better pharmacokinetics and are entering clinical trials to treat syndromes like 8216 [368]. Additional inhibitors are ferrostatin-1 (Fer-1), edaravone, and liproxstatin-1 (Lip-1), which can directly prevent lipid peroxidation, exerting antioxidant effects for neuroprotection in preclinical models against brain ischemia and neurodegeneration [369]. Other strategies are focused on the target antioxidant system (e.g., up-regulation of GPX4 or system Xc-) or enzymes that limit lipid metabolism, such as ACSL4 and LOX, which extend the therapeutic opportunity of diseases that feature necroptotic cell death. Nevertheless, clinical translation remains in its infancy as problems like short half-life, low oral bioavailability, and off-target effects persist [369].

#### Key translational challenges

The development of ferroptosis-targeted therapeutics has several challenges in the translation from bench to bedside. To begin with, the lack of effective biomarkers in clinical samples hinders patient selection and the real-time monitoring of response to therapy [370]. Genetics and heterogeneity affect susceptibility to ferroptosis inducers, further complicating the design of individualized treatments [371]. Drug resistance, particularly in the context of sorafenib and other small-molecule inducers, poses a significant hurdle, requiring combined therapies and novel agents to curtail adaptive resistance mechanisms [372]. There are serious safety concerns about the possible inhibition of ferroptosis or systemic induction in non-target tissues that may cause unintended toxicities, including neurodegeneration or organ dysfunction [373]. Thus, it becomes essential to have validated mechanism-based clinical endpoints and well-characterized preclinical models that are critical for successful clinical translation.

#### Clinical pipeline and future trial design

The clinical pipeline for ferroptosis-targeted therapies is expanding, with more than ten key therapies currently in various stages of clinical trials worldwide (Table 2). Companies such as Kojin Therapeutics, PTC Therapeutics, MitoImmune Therapeutics, Prothegen, Tharimmune, and Sumitomo are leading efforts to develop both inducers and inhibitors for oncology, neurodegeneration, and inflammatory diseases. Regulatory agencies have granted priority designations (e.g., Orphan and Fast Track) to several candidates, reflecting their potential to address unmet medical needs. Future trial designs are focusing on combination regimens (e.g., ferroptosis inducers plus immunotherapy), biomarker-driven patient stratification, and adaptive protocols to optimize efficacy and minimize toxicity [374]. Developing companion diagnostics and liquid biopsy strategies on ferroptosis biomarkers will likely contribute to personalized treatment and real-time tracking [373]. Also, computational modeling and machine learning are becoming more prevalent in designing trials to provide predictions about the best drug combinations or maximum toxicity levels, hypothetically expediting preclinical translation into a clinical trial.

**Table 2:** Drugs modulating ferroptosis under clinical trial

Drug Name	Mechanism/Target	Clinical Status	Indications / Use	Clinical Trial Number(s)	Notes
Deferoxamine (DFO)	Iron chelator, inhibits ferroptosis by chelating Fe3 + and upregulating antioxidants	Approved, ongoing research	Iron overload, neuroprotection	NCT00777140	Widely used iron chelator, studied in spinal cord injury repair (animal models)
Deferiprone (DFP)	Oral iron chelator, ferroptosis inhibitor	Clinical trials	Friedreich Ataxia, Parkinson's Disease	NCT01539837	Pilot clinical trial in Friedreich ataxia, iron removal, MRI monitored
Deferasirox (DFX)	Oral iron chelator	Clinical trials	Iron overload, myelodysplastic syndrome (MDS)	NCT01033747	Proven safe and effective in managing serum ferritin in MDS
CN128	Novel oral iron chelator	Phase IIb clinical trials	β-thalassemia with severe iron overload	NCT05355766	Ongoing long-term efficacy and safety study
Sorafenib (SRF)	Kinase inhibitor, induces ferroptosis by promoting TRIM54-mediated FSP1 ubiquitination and degradation	Approved, cancer therapy	Hepatocellular carcinoma (HCC)	NCT00105443	FDA-approved drug with roles in ferroptosis induction
Lanperisone	Suppresses system Xc-, triggers ferroptosis	Investigational	Acute non-specific low back pain	EudraCT 2021–006042-13	Clinical trial with tolperisone (related compound) for pain, no direct ferroptosis trial info

This table summarizes selected pharmacological agents that modulate ferroptosis and have advanced to clinical evaluation, categorized by their primary mechanism of action, clinical status, and therapeutic indications. Ferroptosis inhibitors include iron chelators—such as deferoxamine (DFO), deferiprone (DFP), and deferasirox (DFX)—which reduce the labile iron pool available for Fenton chemistry, as well as the novel oral chelator CN128, which is under evaluation for iron overload disorders. Ferroptosis inducers include sorafenib, a multikinase inhibitor approved for hepatocellular carcinoma that promotes ferroptosis through TRIM54-mediated FSP1 ubiquitination and degradation, and lanperisone, which suppresses System Xc⁻ activity. The table includes information on clinical trial status, primary indications, and relevant trial registration numbers (NCT or EudraCT identifiers) where available. Agents are grouped according to their primary ferroptosis-modulating mechanism, though several compounds exhibit pleiotropic effects beyond ferroptosis. This compilation reflects the current translational landscape and underscores the growing clinical interest in ferroptosis-targeted therapies for oncology, neurodegenerative disorders, ischemic diseases, and iron overload syndromes. Abbreviations: *DFO* deferoxamine, *DFP* deferiprone, *DFX* deferasirox, *FSP1* ferroptosis suppressor protein 1, *HCC* hepatocellular carcinoma, *MDS* myelodysplastic syndrome, *MRI* magnetic resonance imaging, *TRIM54* tripartite motif-containing protein 54

#### Clinical research progress

Over the years, there have been tremendous developments in clinical and translational research on ferroptosis. Early-phase clinical trials are evaluating the safety and efficacy of ferroptosis inducers in solid tumors, with some agents showing promising anti-tumor activity [199]. Other inhibitors, DFO and edaravone, have been advanced to clinical applications or late-phase testing in neurodegenerative disorders and ischemic diseases, which provide neuroprotective and organ-protective effects [198]. Preclinical research is progressing to identify new drug targets and improve the delivery method (e.g., nanotechnology, cell therapy) and combination therapy with the standard of care [351]. Although these developments have been made, the majority of clinical experience is associated with the initial-phase trials, and additional studies are required before efficacy, safety, and optimum usage within broad patient demographics can be determined [375].

## Conclusion and future perspectives

In summary, this review has unraveled the complexities involved in the relationship between ferroptosis and reproductive dysfunction, highlighting its importance in the etiology of many reproductive disorders. The evidence presented underscores the contributions of iron dysregulation, reactive oxygen species-induced oxidative stress, and lipid peroxidation to the initiation of ferroptosis, which consequently results in reproductive dysfunction. Besides, the relationship between ferroptosis and reproductive hormones, as well as its role in embryonic development and implantation, was defined. This comprehensive overview of the ferroptosis-reproductive dysfunction axis has huge implications for the newer therapeutic strategies for reproductive pathologies. Subsequent research should be oriented toward translating such mechanisms to clinics using ferroptosis-modulating interventions and clarifying molecular mechanisms involved in this complex interplay. Ultimately, this knowledge will empower clinicians and researchers to address the unmet needs of those affected by reproductive disorders.

## Data Availability

N/A.
